# Airborne microbes in five important regions of Chinese traditional distilled liquor (*Baijiu*) brewing: regional and seasonal variations

**DOI:** 10.3389/fmicb.2023.1324722

**Published:** 2024-01-09

**Authors:** Yan Xu, Xue Qiao, Lei He, Wujie Wan, Zhongjun Xu, Xi Shu, Cheng Yang, Ya Tang

**Affiliations:** ^1^College of Architecture and Environment, Sichuan University, Chengdu, China; ^2^Institute of New Energy and Low-carbon Technology, Sichuan University, Chengdu, China; ^3^School of Carbon Neutrality Future Technology, Sichuan University, Chengdu, China; ^4^College of Life Sciences, Sichuan Normal University, Chengdu, China

**Keywords:** fungi, bacteria, seasonal changes, brewing ecosystems, strong-flavor *Baijiu*

## Abstract

*Baijiu* is one of the six primary distilled spirits in the world. It is produced through the solid-state fermentation of grains in the open environment, so high-quality *Baijiu* brewing largely depends on terrior. Environmental microbes are one of the most important factors affecting the quality, quantity, and flavors of *Baijiu*. As atmosphere is a pool and transport pathway for microbes from the ambient environment to *Baijiu* brewing ecosystems, we explored the functional microbes of *Baijiu* brewing in five important regions. The regions fell into two topographical types, namely, plain and river-valley. In total, 41 functional microbes were identified rich (relative abundance >0.1%) in at least one of the regions, such as the fungi of *Aspergillus, Candida, Cladosporium, Debaryomyces, Penicillium, Pichia, Rhizopus, Saccharomyces,* and *Wickerhamomyces* and the bacteria of *Acetobacter, Bacillus, Clostridium, Enterobacter, Lactobacillus, Methanosarcina, Methanobacterium, Methanobrevibacter,* and *Pseudomonas*. However, some functional bacteria (e.g., *Clostridia, Gluconacetobacter,* and *Weissella*) and fungi (e.g., *Dekkera, Eurotium, Issatchenkia*, *Mucor,* and *Phoma*) were not rich or were not detected in the atmosphere. Airborne microbiomes and the Phylogenetic Diversity (PD) index were significantly different between the main brewing season (winter) and the summer break in each region, except for the fungi in one region. In winter, airborne microbiomes were significantly different among almost all the regions. The relative abundance of bacterial fermentation function in each region increased from summer to winter. The relative abundances of fungal yeast function were higher in winter for the plain regions but were higher in summer for the river-valley regions. In sum, our results suggested that: (1) atmosphere was one but not the sole important source of functional microbes for *Baijiu* brewing and (2) microbiomes in different regions might be quite different but they could share some major functions related to *Baijiu* brewing.

## 1 Introduction

*Baijiu* is one of the six primary distilled spirits worldwide, along with brandy, whisky, vodka, rum, and gin. *Baijiu* has a history probably since China’s Jin Dynasty (12th to 13th century) (Li and Chu, 2019), and in modern times it is often used on important Chinese economic and social events. In addition, many Chinese consume *Baijiu* in small quantities on a daily basis (Zheng and Han, 2016), as *Baijiu* has over 100 health-promoting compounds (Du et al., 2023; Hong et al., 2023; Kang et al., 2023). In 2020, the total production of *Baijiu* in China was 10.7 billion liters and gained a sales revenue of US$ 90.3 billion (Tu et al., 2022).

Ethanol and water together contribute to 98% weight of *Baijiu*, with ethanol usually accounting for 38 to 65% of the total volume (Tu et al., 2022). However, the quality, flavor, and aroma of *Baijiu* are primarily determined by the trace components that contribute to the rest 2% weight (Tu et al., 2022). By 2019, over 2,000 species of trace components have been identified, such as aldehydes, acetals, acids, esters, ketones, furans, nitrogen compounds, terpenes, and sulfides (Wang et al., 2015; Hong et al., 2023). Owing to the different trace component profiles among products, *Baijiu* is categorized into three major and nine minor flavor types (Wei et al., 2020; Xu Y. et al., 2022). Among these 12 types, the strong-flavor, also known as Luzhou-flavor and Nongxiang-flavor, dominates 60–70% of China’s *Baijiu* consumption and is characterized by its intense aroma and sweetness mainly from ethyl hexanoate (Xu et al., 2017; Wei et al., 2020; Zhang et al., 2020; Hu et al., 2022).

**Figure 1:**
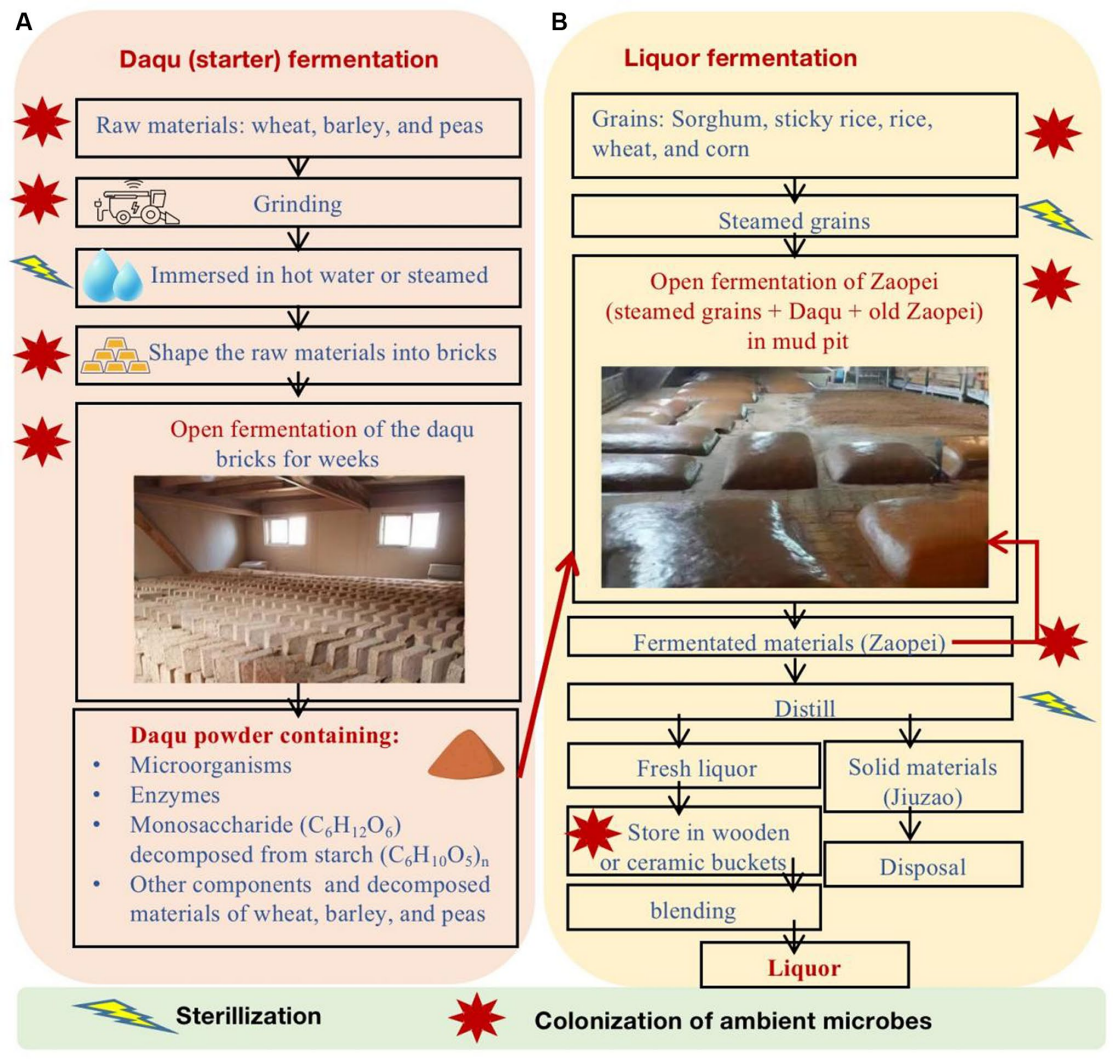
The major production processes of strong-flavor *Baijiu*, including **(A)** Daqu fermentation and **(B)** liquor fermentation.

The strong-flavor *Baijiu* is produced through spontaneously solid-state fermentation of grains in the open environment (Figure 1). The brewing comprises three primary stages: Daqu (starter) fermentation, liquor fermentation, and liquor distillation. Daqu is comprised of wheat, barley, and/or peas and is shaped into bricks. In an open environment, Daqu bricks undergo natural inoculation of microbes from the ambient environment. The microbes in Daqu consume grains and generate enzymes. As a result, matured Daqu provide functional microbes, enzymes, and the precursors of aromatic substances for liquor fermentation. During the liquor fermentation, Daqu and steamed grains (e.g., sorghum, sticky rice, rice, wheat, and/or corn) are mixed (called Zaopei in Chinese) and they are fermented together in an anaerobic condition in mud pits for weeks. Then, the fermented Zaopei are distilled for fresh liquor. Fresh liquor is stored for aging and then is blended for *Baijiu* products. More details of *Baijiu* brewing processes can be found in previous review papers (Zheng and Han, 2016; Jin et al., 2017; Xu et al., 2017; Wei et al., 2020; Yan et al., 2021; Ma et al., 2022; Xu S. et al., 2022; Jin et al., 2023).

Microbes greatly affect the quality and quantity of strong-flavor *Baijiu* (Ma et al., 2022). The functional microbes in the Daqu and liquor fermentation processes are from the ambient environment, pit mud, tools, and others (Figure 1B). Previous studies have found that the major functional microbes in Daqu fall into four categories (Luo et al., 2020; Tu et al., 2022), including mold (e.g., *Aspergillius, Monascus, Mucor, Penicillium,* and *Rhizopus*), yeast (e.g., *Candida, Hansenula, Saccharomyces,* and *Wickerhamomyces*), bacteria (e.g., acetic and lactic acid bacteria and *Bacillus*), and actinomycetes (e.g., *Thermoactinomyces*). The major phyla and genera of microbes in pit mud and Zaopei can be found in previous studies (Luo et al., 2020; Sakandar et al., 2020; Ma et al., 2022; Tu et al., 2022; Xu S. et al., 2022).

Atmosphere serves as one of the microbial pools for Daqu, mud pit, and Zaopei, as shown in Figure 1 and previous studies (Li et al., 2022; Tan et al., 2022; Wang, 2022; Zhou et al., 2024). Local land cover and climate play vital roles in shaping the composition, abundance, dispersal, and seasonal fluctuations of airborne microbes, as airborne microbes primarily originate from local sources (e.g., soil and plants) rather than long-range transport (Zhai et al., 2018). In addition, microbes in indoor air are largely affected by outdoor air (Adams et al., 2013; Meadow et al., 2013). However, the studies on airborne microbes for strong-flavor *Baijiu* brewing are very limited and previous studies are based on traditional isolation and culture technologies (Cao, 1986; Wu, 2004; Lei et al., 2022). A large number of environmental microbe species are difficult to be isolated and cultured (Kaeberlein et al., 2002). Therefore, there is a need to use culture-independent methods to better investigate if the atmospheres of strong-flavor *Baijiu* brewing regions have rich functional microbes in the major brewing season (winter).

**Figure 2:**
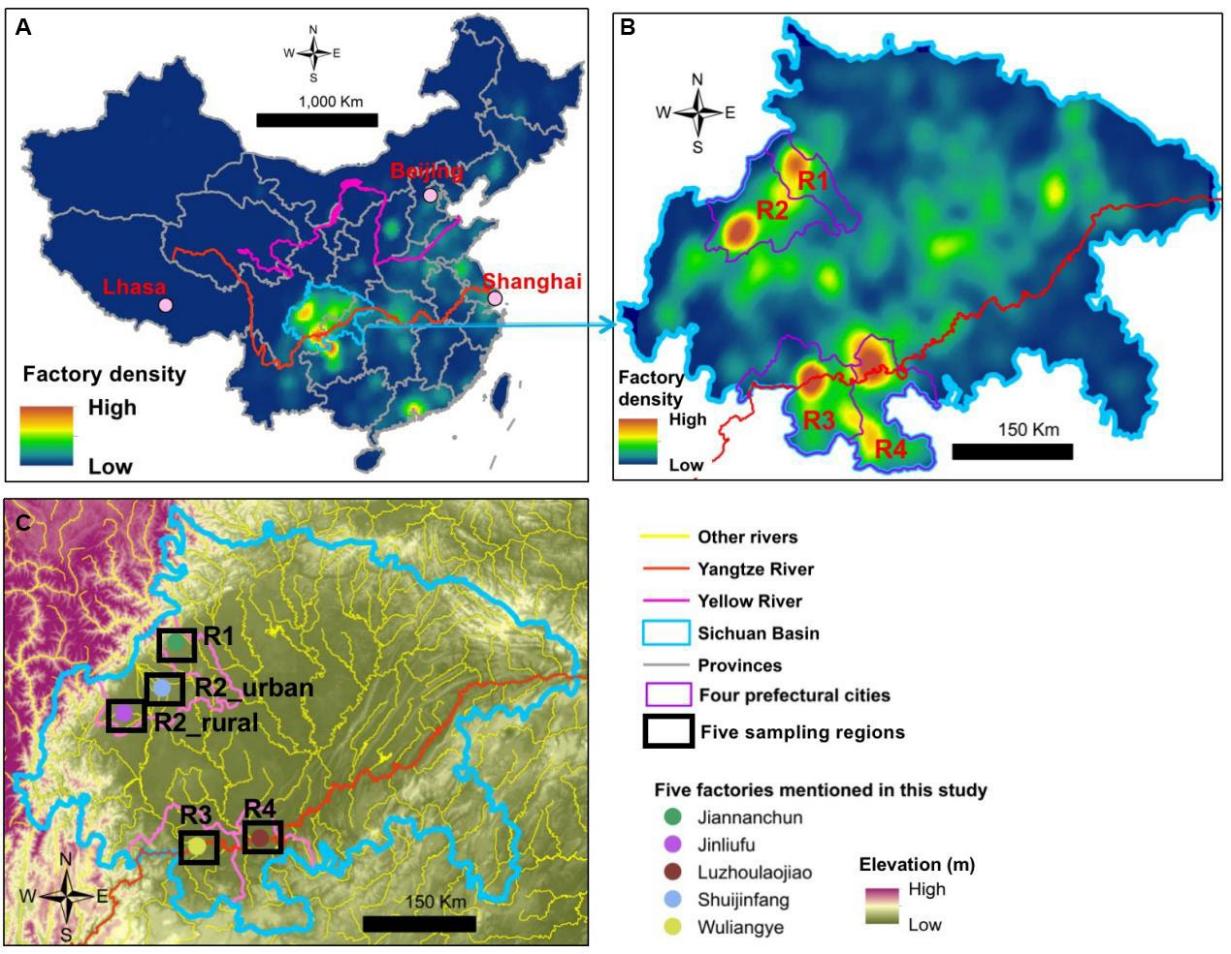
Density maps of *Baijiu* brewing factories in **(A)** China and **(B)** Sichuan Basin and **(C)** the five sampling areas of this study.

The traditional brewing processes and flavors of *Baijiu* are geographically depended (Tan et al., 2022). Sichuan Basin is one of the primary areas of *Baijiu* brewing in China, particularly for the strong-flavor *Baijiu* (Figure 2A). In 2021, the basin approximately contributed to 52% of China’s strong-flavor *Baijiu* production (Hu et al., 2022). In addition, according to the 5th National *Baijiu* Tasting Event in 1989, nine of the 17 national premium brands and 24 of the 53 national high-quality brands belong to the strong-flavor *Baijiu*. Among these 33 national premium/high-quality strong-flavor brands, 16 are from the Sichuan Basin, such as Wuliangye, Luzhoulaojiao, Shuijingfang, and Jiannanchun. Within the basin, four brewing centers exist and they approximately fall into two topographical types, namely, plain and river-valley (Figures 2B,C). Regions 1 and 2 (R1 and R2) are located in the Chengdu Plain, which has an agricultural cultivation history for over 4,500 years. The other two (R3 and R4) are in the river-valleys of Yangtze River.

The major objectives of this study are: (1) to investigate the airborne microbes in five important regions of strong-flavor *Baijiu* in the Sichuan Basin using the high-throughput sequencing (HTS) and (2) to compare airborne microbes between the main brewing season (winter) and the summer break and among different regions. The hypotheses include that: (1) the atmospheres in the five regions have rich functional microbes and (2) the airborne microbial communities have evident seasonal and regional variations. The results of this study will be helpful for the understanding and preservation of this Chinese traditional wisdom about utilizing nature.

## 2 Methods and materials

### 2.1 Sample collection

Airborne microbe samples were collected in five regions during summer (August to September 2021) and winter (December 2021 to January 2022). The five regions covered all the four major *Baijiu* production areas in the Sichuan Basin (Figures 2B,C). Because each of the four *Baijiu* production areas were large and had many *Baijiu* factories, the samples were collected near five famous factories of strong-flavor *Baijiu*, all of which (except Jinliufu) had a history for over 300 years. Jinliufu is a brand born in 1996 but the R2_rural region has a liquor brewing history for over 200 years. In total, 138 samples were collected at 69 sites in the two seasons (Figure 2; Supplementary Figure S1). The coordinates and elevations of each site are listed in Supplementary Table S1.

Three bio-aerosol samplers (ZR-2000 series, Qingdao Junray Intelligent Instrument Co., Ltd) were used in the summer and winter sampling campaigns. Specifically, the sampling heads were placed at a height about 1.8 above the ground and each sampling lasted for 4–6 h during the daytime. Sterile membrane filters (0.22 μm pore size, 47 mm diameter, Polytetrafluoroethylene (PTFE), Jinlong Company) were used and they were stored in sterilized bags individually before sampling. To avoid contamination, the filter holders and the tools used for moving filter papers were all treated with 75% ethanol before each sampling. The measurements of blank samples suggested that contamination during the entire monitoring was negligible. Before DNA extraction, the samples were stored at −80°C.

### 2.2 DNA extraction and PCR amplification

In the lab of Rhonin Biotechnology Company, the filters with samples were cut into small pieces for DNA extraction, and a parallel extraction procedure was performed with the blank filters to check for contamination. The Zymo Research BIOMICS DNA Microprep Kit (Cat# D4301) was used for genomic DNA (gDNA) purification. The integrity and concentration of gDNA were assessed using 0.8% agarose gel electrophoresis and the Tecan F200 instrument, respectively.

In the PCR amplification process for fungi, the fragments of ITS2 region were amplified from the gDNA by using PCR with the primers ITS3 (5′-GATGAAGAACGYAGYRAA-3′) and ITS4 (5′-TCCTCCGCTTATTGATATGC-3′). For bacteria, the 16S rRNA V4 region was amplified by using the primers 515F (5′-GTGYCAGCMGCCGCGGTAA-3′) and 806R (5′ -GGACTACHVGGGTWTCTAAT-3′). The PCR enzyme was TOYOBO KOD-Plus-Neo DNA Polymerase (KOD-401B). The PCR equipment was Applied Biosystems® PCR System 9,700. For each sample, the PCR solution (50 μL) contained: 1X PCR Buffer for KOD-Plus-Neo, 0.2 mM dNTPS, 1.5 mM MgSO_4_, KOD-Plus-Neo (1 U/50 μL), 20 ng/μL DNA template, 0.3 μM U515F (for bacteria), 0.3 μM U806R (for bacteria), 0.3 μM ITS3 (for fungi), 0.3 μM ITS4 (for fungi), and 31 μL H_2_O.

The PCR thermal cycling profile was 94°C for 1 min; 25–30 cycles of 94°C for 20 s, 54°C for 30 s, and 72°C for 30 s; 72°C for 5 min; hold at 4°C. Triplicates were performed for each sample. After the PCR amplification, the triplicates were mixed as one sample. The final PCR products were separated by 2% agarose gel electrophoresis and recovered by the Zymoclean Gel Recovery Kit (D4008). The amplicons were quantified by the Qubit^@^ 2.0 Fluorometer (Thermo Scientific) and pooled with equal molar amounts. Sequencing libraries were generated using the NEBNext Ultra II DNA Library Prep Kit for Illumina (NEB#E7645L; New England BioLabs Company). Sequencing was performed on an Illumina MiSeq instrument (Illumina, San Diego, CA, United States) with the Hiseq Rapid SBS Kit v2 (FC-402-4023 500 Cycle) and the PE250 sequencing method.

### 2.3 Sequence and statistical analyses

After high-throughput sequencing, the FLASH software (version 1.2.11) was used to assemble the paired-end files. Sequence quality filtering was performed using QIIME2 (version 2020.2) and the sequences with length shorter than 200 bp were removed. Then, the Amplicon Sequence Variants (ASVs) were generated, and the taxonomic information were assigned to all fungal ASVs using the Unite database (version 8.3) and to all bacterial ASVs using the SILVA database (version 138). Finally, the files of operational taxonomic units (OTUs), taxonomy, and evolutionary tree were obtained and were further used in statistical analyzes. The rarefaction curve of each sampling region in each season approached an asymptote (Supplementary Figure S2), suggesting that most of the OTUs/species of fungi and bacteria in the atmosphere were observed.

The microbial compositions and functions were calculated and then compared among regions and between seasons. Specifically, the R software (version 4.0.5) with the vegan, ggplot2, pheatmap, stats, GuniFrac, Picante, and ape packages were used to calculate and visualize the alpha and beta diversity indices and the microbial compositions for each sampling region in each season. Then, these diversity indices and the major genera were compared among regions and between seasons. Also, the seasonal and regional variations in microbes were investigated by using the Principal Coordinate Analyzes (PCoA) with the Jaccard distances. The genera had higher contributions to the seasonal and spatial variations were identified by using the LefSe method (i.e., Line Discriminant Analysis (LDA) Effect Size; https://bitbucket.org/biobakery/biobakery/wiki/Home). In this study, only the genera with LDA scores greater than 4 and having relative abundances larger than 3% were presented. The functions of bacteria and fungi were predicted using the FAPROTAX (Louca et al., 2016) and FUNGuild (Nguyen et al., 2016), respectively.

## 3 Results

To better understand the spatio-temporal variations, the microbial data were categorized into ten groups, including R1_S (Region 1, summer), R1_W (Region 1, winter), R2_rural_S (the rural area of R2, summer), R2_rural_W, R2_urban_S (the urban area of R2, summer), R2_urban_W, R3_S, R3_W, R4_S, and R4_W.

### 3.1 Microbial composition

**Figure 3:**
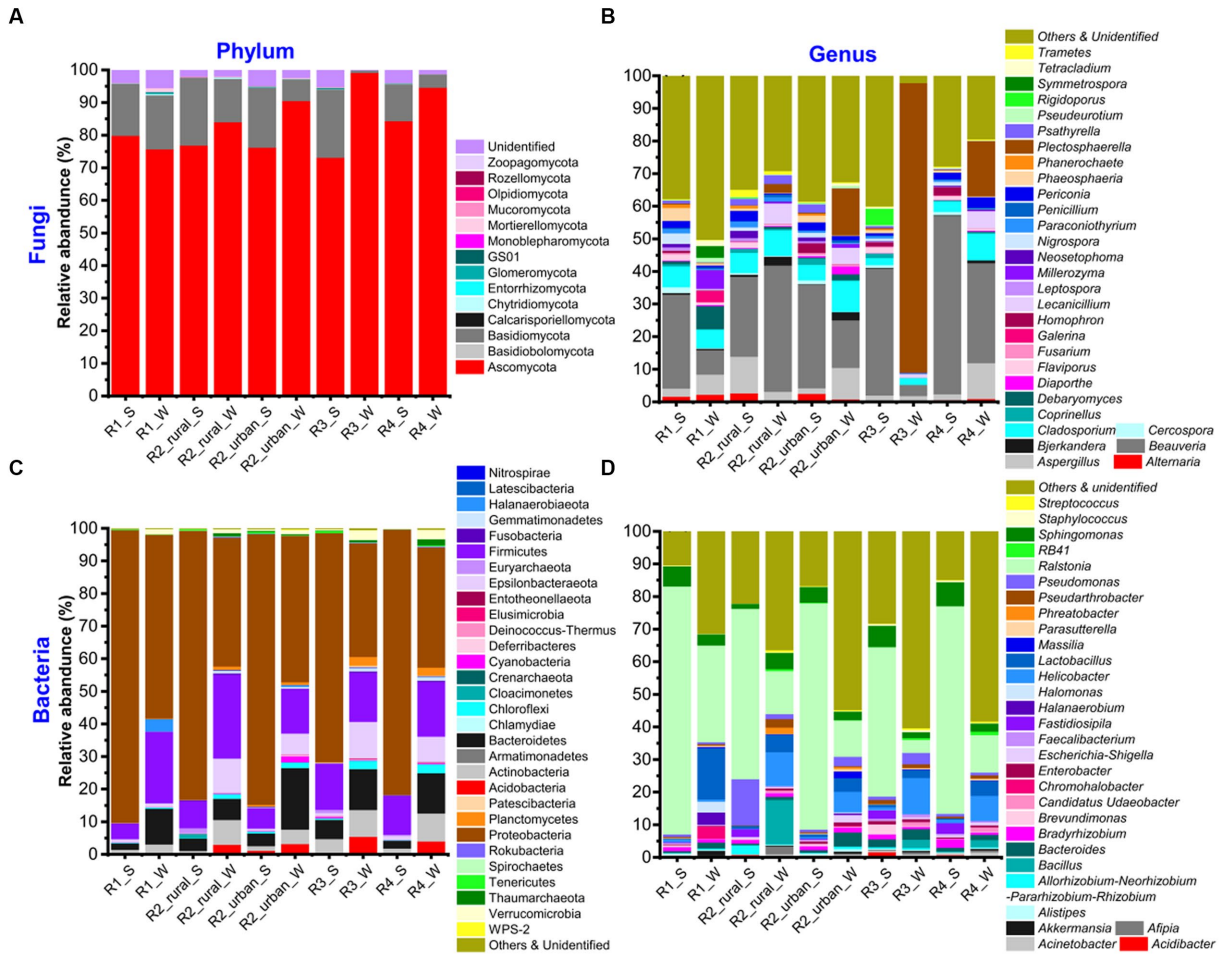
The relative abundances of **(A, B)** fungi and **(C, D)** bacteria at the phylum and genus levels in the five sampling regions in summer (S) and winter (W).

Supplementary Table S2 shows the total numbers of phyla and genera identified for each group, while Supplementary Tables S3–S6 present the phyla and genera of fungi and bacteria. The top 30 richest genera and their seasonal variations are presented in Figure 3 and Supplementary Figure S3, respectively.

#### 3.1.1 Fungi

The identified fungi belonged to 5–9 phyla and 145–419 genera in each group (Supplementary Table S2). Ascomycota was the most abundant phylum in all the groups, having a relative abundance about 75–95% (Figure 3; Supplementary Table S3). The other phyla rich in at least one group included Basidiomycota (0.71–21%), Chytridiomycota (0–0.38%), GS01 (0–0.18%), Mortierellomycota (0–1.16%), and Mucoromycota (0.01–0.14%). The unidentified phyla had a total abundance of 0.1–5.5% in each group. At the genus level, there were five major characteristics (Figure 3; Supplementary Table S4). Firstly, the top 30 richest genera were generally similar but with varying abundances among the ten groups. *Beauveria* was the richest in all the groups except for R3_W and had a relative abundance of 7.5–54% in each group. Secondly, the richest genus of R3_W was *Plectosphaerella* (89%). Thirdly, the abundances of all the genera in R1_W were ≤ 7.5%. Fourthly, in R2_urban_W, *Plectosphaerella*’s richness (14%) was close to that of *Beauveria* (15%). Fifthly, the unidentified and poor (abundance <0.1%) genera together contributed to large portions (12–26%) in all the groups except for R3_W. The portion in R3_W was about 1%.

#### 3.1.2 Bacteria

There were 34–45 phyla and 411–820 genera identified in each group (Supplementary Table S2). The top 30 genera were similar but with varying abundance among the ten groups (Figure 3; Supplementary Table S5). The most abundant phylum was always Proteobacteria (35–90%). The other phyla with abundances larger than 10% in at least one group were Bacteroidetes (2–19%), Epsilonbacteraeota (0.2–11%), and Firmicutes (5–26%). The other phyla that had abundances over 1% in at least one group were Actinobacteria, Acidobacteria, Chloroflexi, Cloacimonetes, Cyanobacteria, Euryarchaeota, Gemmatimonadetes, Planctomycetes, Thaumarchaeota, and Verrucomicrobia. At the genus level, *Ralstonia* was the richest (11–76%) in all the groups except for R3_W (Figure 3; Supplementary Table S7). In R3_W, the richest genus was *Helicobacter* (11%) and the abundance of *Ralstonia* was 4%. The other genera with abundances larger than 10% in at least one group were *Bacillus*, *Lactobacillus,* and *Pseudomonas*.

### 3.2 Spatio-temporal variations in the microbiomes

#### 3.2.1. Alpha diversity

**Figure 4:**
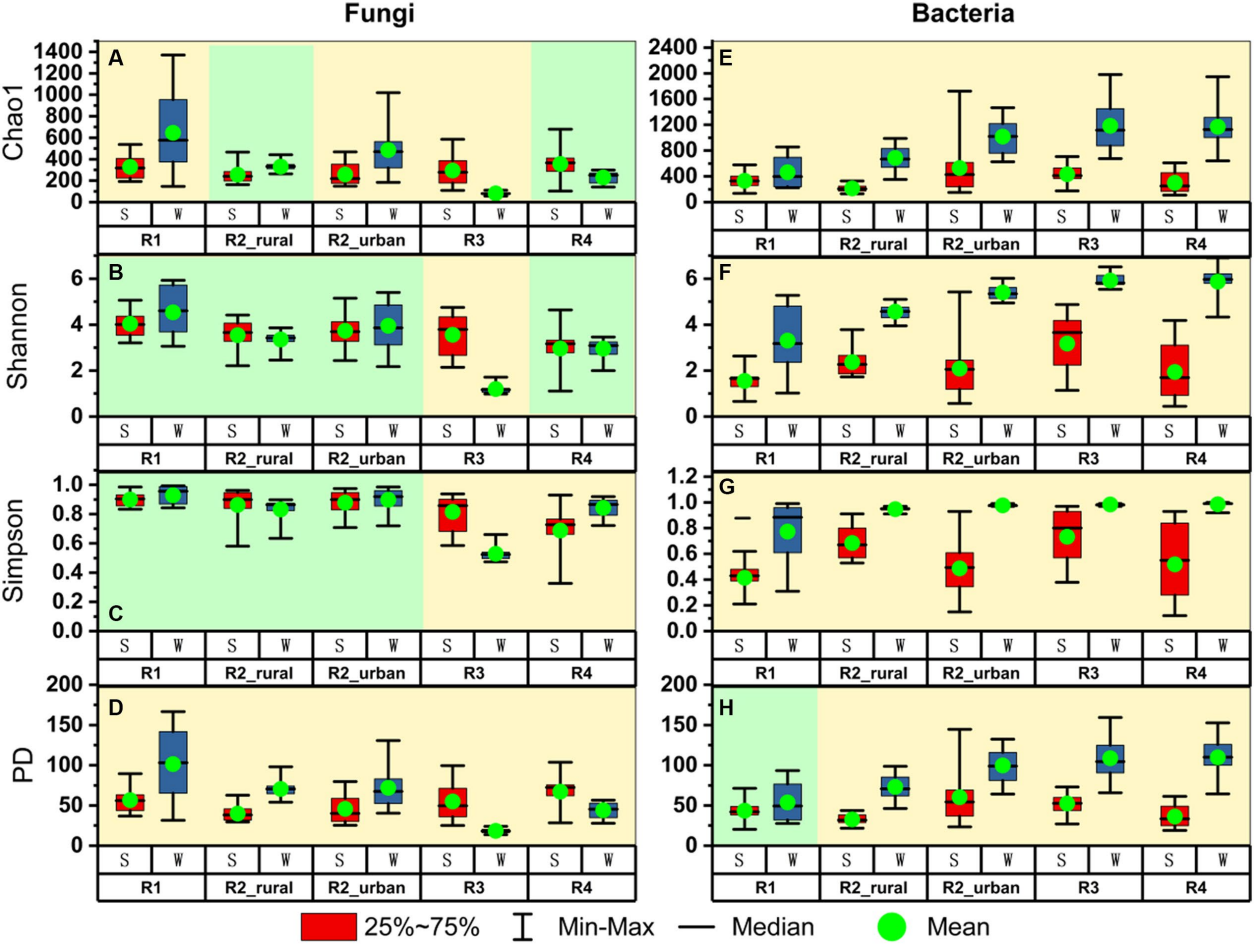
The comparisons of alpha diversity between summer (S) and winter (W) for **(A–D)** fungi and **(E–H)** bacteria in each region.

Figure 4 presents the alpha diversity indices for each group. Among the four indices, Chao1 estimates species’ richness, while the Shannon and Simpson indices consider not just richness but also evenness and dominance. The Phylogenetic Diversity (PD) index incorporates phylogenetic relationships to evaluate the evolutionary diversity within a community (Faith, 1992).

There were three major seasonal variation patterns in the alpha diversity (Figure 4). Firstly, all the indices of bacteria except for PD in R1 were significantly higher in winter than in summer (*p* < 0.05). In R1, the mean value of PD in winter was higher than that in summer but the difference was insignificant (*p* > 0.05). Secondly, the PD values of fungi were significantly different between the two seasons in each region. In the plain regions (including R1, R2_rural, and R2_urban), the fungal PD values were significantly higher in winter than in summer. In contrast, the fungal PD values in the valley regions (including R3 and R4) were significantly higher in summer than in winter. Thirdly, all the four fungal indices in R3 were significantly lower in winter than in summer.

#### 3.2.1. Beta diversity and the major genera contributing to the spatio-temporal variations

**Figure 5:**
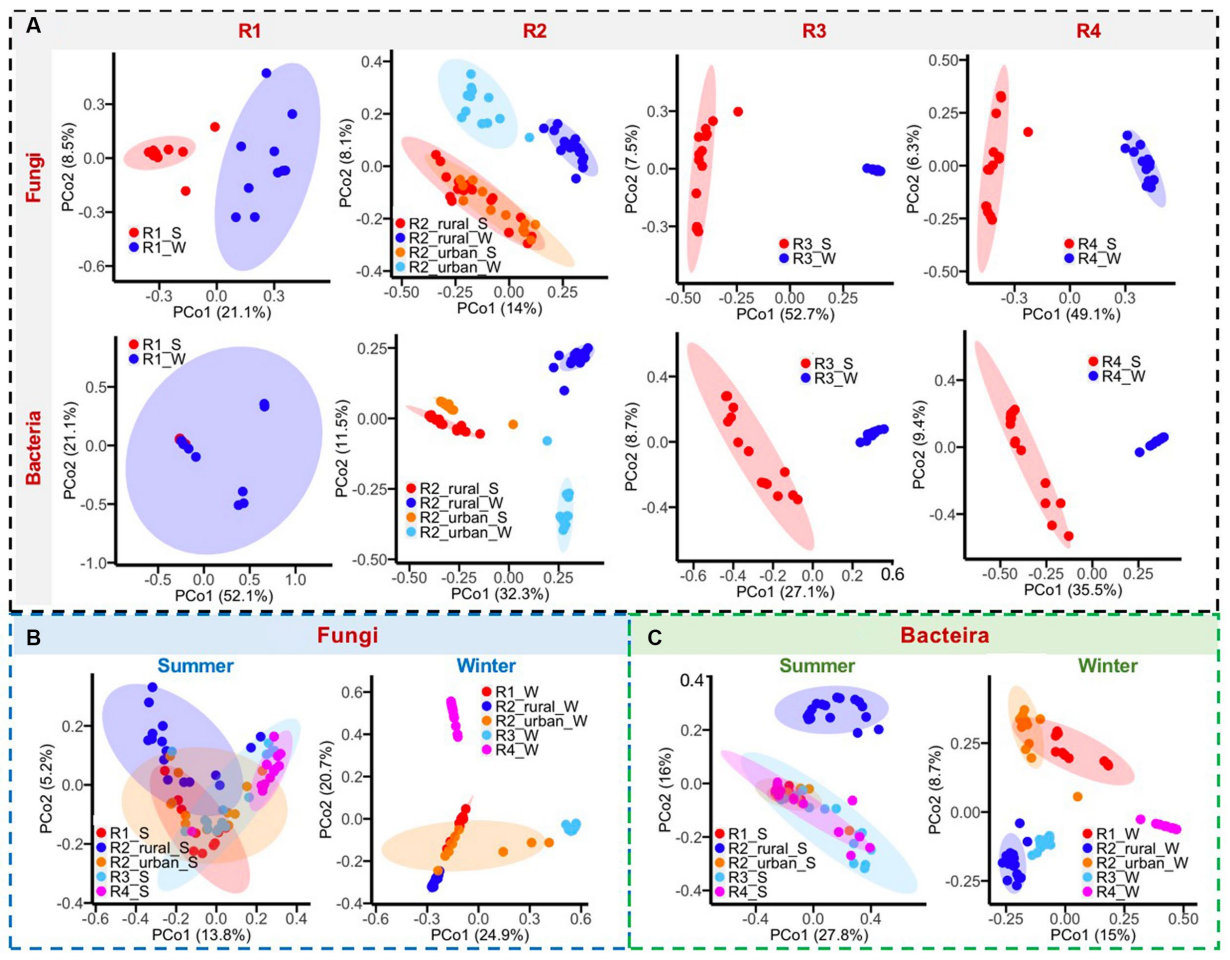
**(A)** Comparisons of bacterial and fungal communities between summer (S) and winter (W), **(B)** comparisons of fungal communities among regions, and **(C)** comparisons of bacterial communities among regions.

Both fungal and bacterial communities were quite different between summer and winter in each region, except for the bacteria of R1 (Figure 5A), the PD values of which were similar between summer and winter (Figure 4H). As shown in Supplementary Figure S4, the fungal genera having higher contributions to the seasonal variations (LDA >4 and relative abundance ≥3%) in at least one region were *Alternaria, Aspergillus, Beauveria, Debaryomyces, Diaporthe, Millerozyma, Nigrospora, Paraconiothynum, Periconia, Plectosphaerella, Psathyrella, Symmetrospora,* and *Rigidoporus. T*he bacterial genera with higher contributions were *Afipia, Allorhizobium-Neorhizobium-Pararhizobium-Rhizobium, Bacillus, Bradyrhizobium, Brevundimonas, Chromohalobacter, Escherichia Shigella, Fastidiosipila, Halanaerobium, Hlicobacter, Lactobacillus, Pseudomonas, Ralstonia,* and *Sphingomonas*.

The differences in microbial communities among regions were greater in winter than in summer (Figures 5B,C). In summer, the fungal and bacterial communities overlapped among the five regions, except for R2_rural_S. In winter, only R1_W and R2_urban_W had considerable overlaps in the fungal and bacterial communities. The fungal genera with larger contributions to the regional differences in at least one region were *Alternaria, Aspergillus, Beaiveria, Cladosporium, Debanyomyces, Lecanillium, Millerozyma, Nigrospora, Plectosphaerlla,* and *Periconia*, while the bacterial genera were *Bacillius, Helicobacter, Lactobacillus, Pseudomonas, Ralstonia,* and *Sphingomonas*.

### 3.3 Spatio-temporal variations in the functional microbes

Table 1 summarizes the major functional microbes of strong-flavor *Baijiu* brewing according to previous studies (Zou et al., 2018; Cheng et al., 2023). Among these microbes, 41 genera were rich in at least one of the ten sample groups. However, some others were not detected in this study, including the fungi of *Brettanomyces, Dekkera, Eurotium, Hansenula, Issatchenkia, Merimbla, Neurospora, Naumovozyma, Sporidiobolus, Torulaspora,* and *Toxicocladosporium* and the bacteria of *Caloramator, Caproiciproduvens, Clostridia, Gluconacetobacter, Olsenella, Porphyromonas, Petrimonas, Petrimonas, Rummeliibacillus, Sedimentibacter, Sporolactobacillus,* and *Spirochaetes SHA-4*.

**Table 1:** The dominant genera of bacteria and fungi reported in the Daqu, pit mud, and Zaopei from the strong-flavor *Baijiu* factories in our sampling regions.^a^

Sample types	Groups	Genera^b^
Daqu	Bacteria	*Acetobacter, Bacillus, Enterobacter, Lactobacillus, Leuconostoc, Pantoea, Staphylococcus, Thermoactinomyces,* and *Weissella*
	Fungi	*Acremonium, Aspergillus, Brettanomyces, Candida, Cerospora, Cladosporium, Hansenula, Dekkera, Lichtheimia, Merimbla, Monascus, Mucor, Neurospora, Paecilomyces, Penicillium, Pichia, Rhizopus, Saccharomycopsis, Sporidiobolus, Talaromyces, Thermoascus, Thermomyces, Wallemia,* and *Wickerhamomyces*
Pit mud	Bacteria^c^	*Acinetobacter, Aminobacterium, Bacteroides, Brevibacillus, Caloramator, Clostridium, Clostridium IV, Caldicoprobacter, Caproiciproduvens, Clostridia, Christensenellaceae R-7, Corynebacterium, Flavobacterium, Lactobacillus, Lysinibacillus, Methanoculleus, Methanosaeta, Methanosarcina, Methanobacterium, Methanobrevibacter, Mycobacterium, Olsenella, Paenibacillus, Porphyromonas, Petrimonas, Proteiniphilum, Pseudomonas, Petrimonas, Rummeliibacillus, Sedimentibacter, Sporolactobacillus, Syntrophomonas,* and *Spirochaetes SHA-4*
	Fungi	*Aspergillus, Candida, Cladosporium, Davidiella, Debaryomyces, Fusarium, Malassezia, Mucor, Phoma, Penicillium, Pichia, Rhizopus, Saccharomyces, Thrichosporon, Thermoascus, Wickerhamomyces, Thermomyces, Toxicocladosporium,* and *Wallemia*
Zaopei	Bacteria	*Acetobacter, Alcaligenes, Bacillus, Gluconacetobacter, Lactobacillus,* and *Prevotella*
	Fungi	*Aspergillus, Candida, Debaryomyces, Eurotium, Issatchenkia, Kazachstania, Naumovozyma, Pichia, Saccharomyces, Torulaspora, Talaromyces, Thermomyces,* and *Thermoascus*

a The genera of this table are collected from Zou et al. (2018), Tu et al. (2022), and Cheng et al. (2023).

b The genera in blue were rich in the atmosphere in at least one group (relative abundance > 0.1%; Supplementary Tables S4, S6). The genera in green were detected in at least one group but with relative abundance < 0.1%.

c The major phyla were Actinobacteria, Bacteroidetes, Euryarchaeota, Firmicutes, and Protecobacteria.

**Figure 6:**
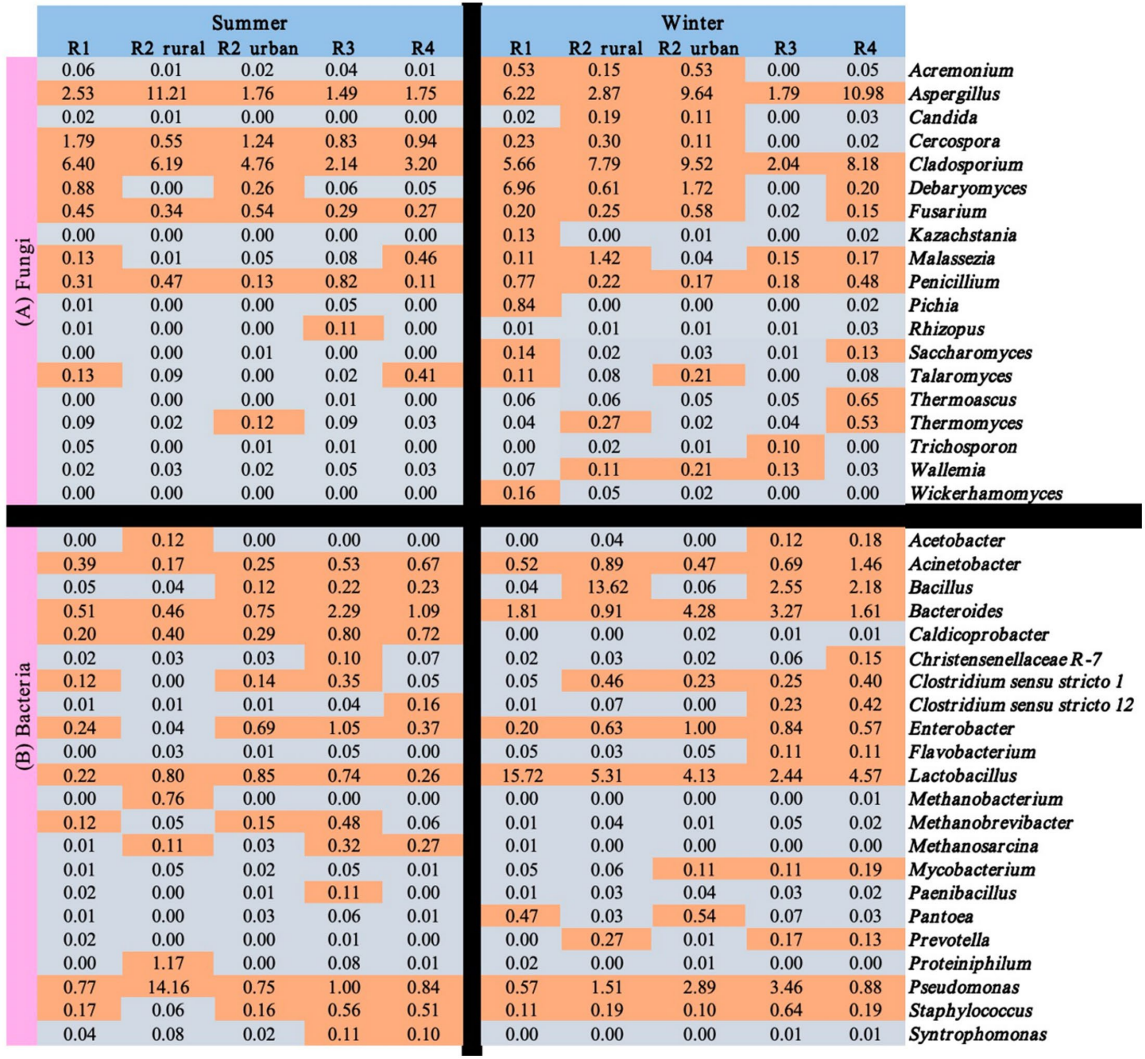
Relative abundances (%) of the functional microbes identified in the atmospheres of the five regions.

Figure 6 shows the seasonal and regional variations of the 41 functional microbes with relative abundance >0.1% in at least one of the ten sample groups. Among the 41 microbes, only three fungal genera (*Aspergillus*, *Cladosporium,* and *Penicillium*) and six bacterial genera (*Acinetobacter, Bacteroides, Enterobacter, Lactobacillus, Pseudomonas,* and *Staphylococcus*) were rich in all the regions in winter. *Rhizopus* and seven bacterial genera (*Caldicoprobacter, Methanobacterium, Methanobrevibacter, Methanosarcina, Paenibacillus, Proteiniphilum,* and *Syntrophomonas*) were poor (<0.1%) in all the regions in winter.

### 3.4 Characteristics of the microbial functions

#### 3.4.1 Major microbial functions

**Figure 7:**
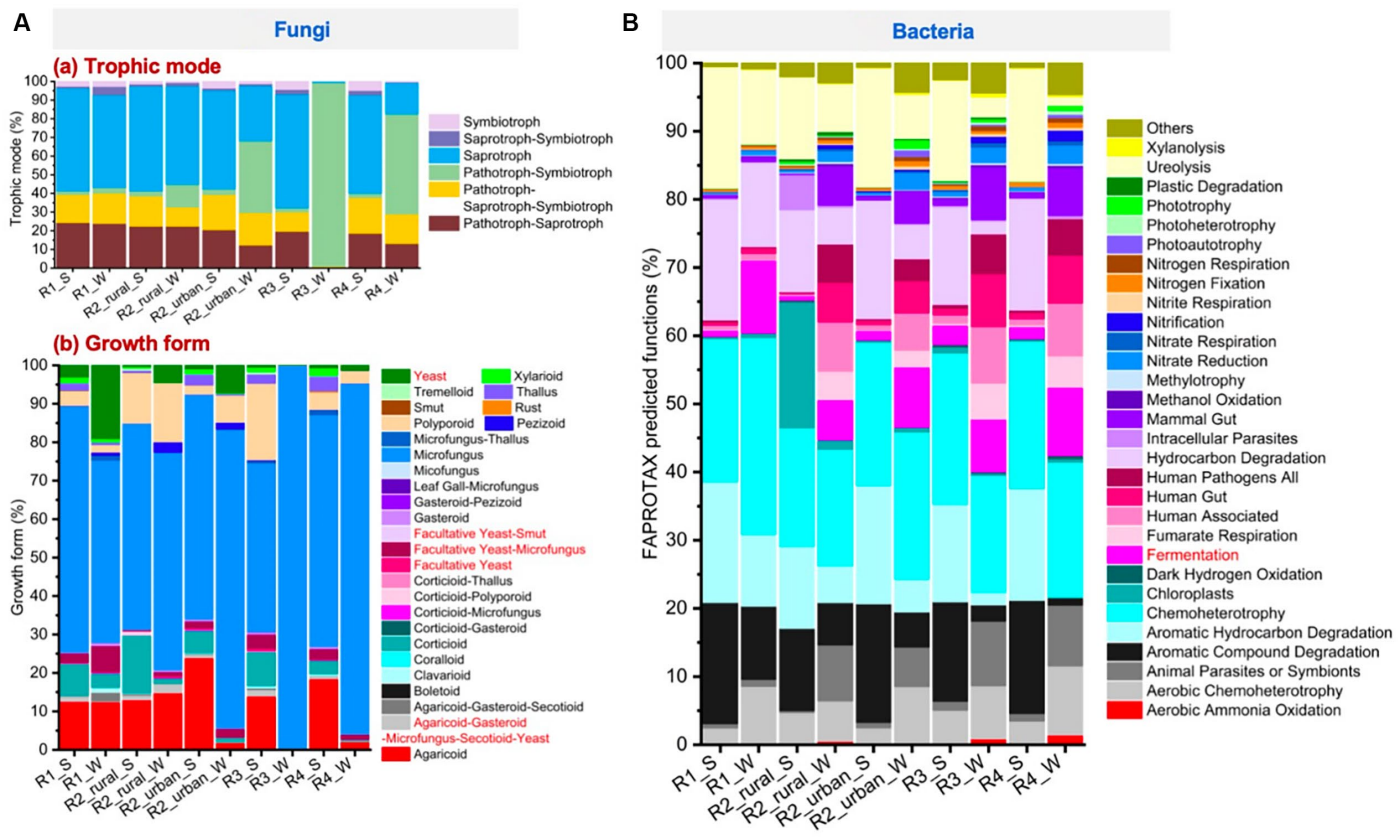
The major functions predicted by using the FUNGuild and FAPROTAX tools for **(A)** fungi and **(B)** bacteria, respectively.

Figure 7A presents the trophic modes and growth forms of fungi. All the groups except for the river-valley regions in winter (R3_W and R4_W) had high abundances of saprotroph (17–56%), pathotroph-saprotroph-symbiotroph (10–19%), and pathotroph-saprotroph (12–24%). Pathotroph-symbiotroph was also large in R2_urban_S (12%) and R2_urban_W (38%), and this mode was the dominant in R3_W and R4_W (98 and 54%, respectively). Pathotroph-symbiotroph accounted for <3% in R1 and rural R2 in both seasons. Regarding growth forms, microfungus was the richest in the ten groups (44–100%), followed by agaricoid (0–24%), corticioid (0–15%), facultative yeast-microfungus (0–7%), polyporoid (0–20%), thallus (0–4%), and yeast (0–19%).

Figure 7B presents the top 30 largest functions identified for bacteria. The largest function for all the groups was chemoheterotrophy (17–29%). Fermentation was 0.6–11% for each group. The other functions that had a relative abundance larger than 10% for at least one group were aerobic chemoheterotrophy, aromatic compound degradation, aromatic hydrocarbon degradation, hydrocarbon degradation, and ureolysis.

#### 3.4.2 Seasonal changes in microbial functions

Significant seasonal changes of fungal yeast and bacterial fermentation were identified, as shown in the heatmaps of Supplementary Figures S5, S6. The bacterial fermentation of each region increased from 0.6–3% in summer to 6–11% in winter. While, the variations in fungal yeast functions were much more complex. In the plain regions (R1 and R2), the abundances of the fungal yeast and facultative yeast-microfungus functions were significantly higher in winter (4.5–19% and 1.1–6.8%, respectively) than in summer (0.3–3% and 0.3–2.4%, respectively). In contrast, in the river-valley regions (R3 and R4), the yeast and facultative yeast-microfungus functions were lower in winter (0.3–1.3% and 0.2–1.1%, respectively) than in summer (0.53–0.71% and 2.7–3.7%, respectively). The abundances of facultative yeast-smut and facultative yeast were always low (<2%) in all the regions. Agaricoud-gasteroid-microfungus-secotioid-yeast was higher in summer for all the regions (0.6–2.4%) except for rural R2.

## 4 Discussion

In this section, the relationships between *Baijiu* brewing and the airborne functional microbes identified in this study will be discussed. Then, the seasonal and regional variations in the airborne microbes and microbial functions will be discussed.

### 4.1 Functional microbes in the atmosphere

Table 1 confirmed that atmosphere was a source of functional microbes for strong-flavor *Baijiu* brewing. Some of the functional bacteria (e.g., *Bacillus, Enterobacter, Lactobacillus, Lactococcus*, *Pseudomonas,* and *Staphylococcus*) and fungi (e.g., *Aspergillus*, *Candida, Cladosporium, Kazachstania, Penicillium, Pichia, Rhizopus,* and *Wicherhamomyces*) were rich in at least one of the ten data groups, but some others were not (e.g., bacteria: *Thermoactinomyces* and *Weissella;* fungi: *Saccharomycopsis* and *Mucor*). This reflected that atmosphere was one but not the sole source of the functional microbes. Previous studies have found that soils, grains, water, air, workers, and brewing tools are all sources of functional microbes (Zhai et al., 2018; Li et al., 2022). For example, indoor ground and dust were one of the main sources of *Bacillus,* Clostridiaceae, *Pichia*, *Pseudomonas*, *Saccharomyces,* and Lactobacillaceae in a factory (Wang, 2022 and references therein). Pit mud used in several factories from R2-R4 were rich in *Methanoculleus, Methanosaeta*, and *Phoma*, all of which were poor in the atmospheres of this study [Figure 6; Table 1; Zou et al. (2018) and references therein].

The microbial species listed in Table 1 can affect the basic processes of *Baijiu* brewing, including saccharification, alcohol fermentation, and aromatic component formation (Zheng and Han, 2016; Jin et al., 2017; Yan et al., 2021). During the saccharification process, especially in Daqu, a variety of extracellular enzymes (e.g., amylase, glucoamylase, protease, cellulose, and lipase) hydrolyze the macromolecules in cereals to form fermentable sugars (Wang, 2022). These extracellular enzymes could be secreted by the species identified in this study, such as the bacteria of *Bacillus, Enterobacter, Lactobacillus, Staphylococcus, Thermoactinomyces,* and *Weissella* and the fungi of *Aspergillus, Cladosporium, Mucor, Penicillium,* and *Rhizopus* (Figure 3; Table 1; Xu et al., 2017; Zou et al., 2018; Wang, 2022; Cheng et al., 2023). Yeast species in the fermentation process can be divided into two groups, which are responsible for ethanol production and flavor compound formation, respectively (Wang, 2022). The species detected in this study and related to ethanol production included but were not limited to S*accharomycopsis, Saccharomyces,* and *Wicherhamomyces* (Wang et al., 2019; Figure 3; Table 1). Esters are the main aromatic components, and lactic acids are one of their precursors (Cheng et al., 2023 and references therein). Lactic acid is mainly generated from carbohydrates by LAB (e.g., *Lactobacillus, Streptococcus, Lactococcus, Leuconostoc,* and *Weissella* in this study). The interactions among *Candida, Lactobacillus, Pichia,* and *Zygosaccharomyces* are likely related to the esters and acids such as hexanoic acid, butyl hexanoate, and ethyl caproate (Tan et al., 2022). *Mucor, Monascus, Bacillus,* and *Lactobacillus* also have been found to generate aromatic components (Wang, 2022 and references therein).

### 4.2 Seasonal and regional variations in the microbiomes and microbial functions

Airborne microbes are affected by the synergistic effect of many factors, such as meteorological parameters, sources, anthropogenic activities, air pollutants, among others. The seasonal and spatial variations in these factors directly and indirectly affect airborne microbiome. Solar radiation mostly has a negative impact on microbes, as ultraviolet radiation can kill microbes. The impact mechanisms of temperature on airborne microbes have not been clearly understood, but previous studies suggest that cold air may facilitate the release and transport of bacteria (Zhai et al., 2018). Rain, high RH, and high wind speed may trigger spore release and/or was beneficial to bacterial release and growth; However, they can also accelerate the deposition or disperse of microbes (Zhai et al., 2018). Air pollutants, such as particulate matter and ozone, affect the disperse, deposition, and growth of microbes. For example, ozone at high concentration is toxic to microbes. Airborne microbes are mainly from local sources, and every region may have their particular and primary sources, such as different plants, soil, animals, and waters (Zhai et al., 2018).

Seasonal changes in airborne microbes are not uniform across the world, considering the temperature, land cover, topography, and anthropogenic activities (Zhai et al., 2018). In this study, the bacterial and fungal communities were quite different between the two seasons in all the regions except for the bacteria in R1 (Figure 5A). The PD richness of bacteria in all the five regions were significantly higher in winter than in summer, and this might be partially explained by the cold air, lower solar radiation, low rainfall, low wind speed, and low ozone pollution in winter. The PD values of fungi presented opposite seasonal changes between the plain and river-valley regions (Figure 4). However, to date, there is no sufficient data to well explain the complicated seasonal variation patterns of microbes for the five regions.

Despite the large regional differences in microbes in winter (Figures 4–6), the major fungal growth forms (e.g., agaricoid, corticioid, microfungus, polyporoid, and the yeast-related) and the major bacterial functions (e.g., aerobic chemoheterotrophy, animal parasites or symbionts, aromatic hydrocarbon/compound degradation, chemoheterotrophy, fermentation, fumarate respiration, human associated, human gut, human pathogens all, hydrocarbon degradation, mammal gut, nitrate reduction, ureolysis, and others) were generally similar among the regions (Figure 7). Particularly, there were some seasonal functional variations that might be beneficial for *Baijiu* brewing in winter. Firstly, the bacterial fermentation function was higher in winter than in summer for all the regions (Figure 7; Supplementary Figure S6). Secondly, the functions of fungal yeast (Figure 7) and sum_yeast_related (Supplementary Figure S5) were higher in winter for the plain regions (R1 and R2) but were not for the river-valley regions. Thirdly, the aromatic compound degradation, aromatic hydrocarbon degradation, and hydrocarbon degradation were lower in winter than in summer for all the regions. All the functions mentioned above may affect *Baijiu* brewing but the impact mechanisms and degrees still need future investigations.

## 5 Conclusion

Based on the observations in five important brewing regions of strong-flavor *Baijiu*, 41 functional microbes were rich in at least one region in summer and/or winter. However, some other functional microbes were poor or even not detected in all the regions in both seasons. In addition, the five regions have distinct airborne microbiomes in winter, but considerable regional overlaps of microbiomes were found during the summer break. There were also some significant changes in microbiomes between summer and winter, as the four alpha diversity indices of bacteria (except the PD of one region) and the PD index of fungi were significantly different between the two seasons. In addition to the seasonal changes in microbial composition, the relative abundance of bacterial fermentation function was higher in winter for all the five regions. The fungal yeast function was higher in winter than in summer for the plain region but lower for the river-valley regions. In conclusion, the above results suggested that: (1) atmosphere was one but not the sole important source of functional microbes and (2) different regions may have different airborne microbiomes but they could share some major functions related to *Baijiu* brewing. Since the mechanisms between *Baijiu* brewing and environmental microbial communities are complex and are hard to quantify, it is important to protect the ambient environments for high-quality *Baijiu*.

## Data availability statement

The data presented in this study can be obtained from the National Center for Biotechnology Information (NCBI) database (PRJNA1052375 and PRJNA1052644).

## Author contributions

YX: Data curation, Methodology, Visualization, Writing – original draft. XQ: Conceptualization, Data curation, Formal analysis, Funding acquisition, Investigation, Supervision, Visualization, Writing – original draft, Writing – review & editing. LH: Conceptualization, Methodology, Resources, Writing – original draft. WW: Data curation, Resources, Writing – original draft. ZX: Data curation, Visualization, Writing – original draft. XS: Data curation, Investigation, Writing – original draft. CY: Data curation, Investigation, Resources, Writing – original draft. YT: Conceptualization, Funding acquisition, Project administration, Resources, Supervision, Writing – review & editing.

## References

[ref1] Adams R. I. Miletto M. Taylor J. W. Bruns T. D. (2013). Dispersal in microbes: fungi in indoor air are dominated by outdoor air and show dispersal limitation at short distances. ISME J. 7, 1262–1273. doi: 10.1038/ismej.2013.28 23426013 PMC3695294

[ref2] Cao D. (1986). The production of Daqu *baijiu* and airborne microorganisms. Liquor-Making Sci. Technolog. 4, 21–23. doi: 10.13746/j.njkj.1986.04.007

[ref3] Cheng W. Chen X. Guo Y. Zhou D. Zeng H. Fu H. (2023). The microbial diversity and flavour metabolism of Chinese strong flavour *baijiu*: a review. J. Inst. Brew. 129, 15–38. doi: 10.58430/jib.v129i1.12

[ref4] Du P. Jiao G. Zhang Z. Wang J. Li P. Dong J.. (2023). Relationship between representative trace components and health functions of Chinese *baijiu*: a review. Fermentation 9:658. doi: 10.3390/fermentation9070658

[ref5] Faith D. P. (1992). Conservation, evaluation and phylogenetic diversity. Biol. Conserv. 61, 1–10. doi: 10.1016/0006-3207(92)91201-3

[ref6] Hong J. X. Zhao D. R. Sun B. G. (2023). Research progress on the profile of trace components in *baijiu*. Food Rev. Intl. 39, 1666–1693. doi: 10.1080/87559129.2021.1936001

[ref7] Hu J. H. Chen Y. Q. Xue X. X. Han X. L. (2022). Overview of development of strong-flavor *baijiu*. China Brewing 41, 24–30.

[ref8] Jin G. Zhu Y. Rinzema A. Wijffels R. Xu Y. (2023). “Process principles and engineering of solid-state fermentation of *baijiu*” in Science and engineering of Chinese liquor (*baijiu*) microbiology, chemistry and process technology (Berlin: Springer), 121–142.

[ref9] Jin G. Zhu Y. Xu Y. (2017). Mystery behind Chinese liquor fermentation. Trends Food Sci. Technol. 63, 18–28. doi: 10.1016/j.tifs.2017.02.016

[ref10] Kaeberlein T. Lewis K. Epstein S. S. (2002). Isolating “uncultivable” microorganisms in pure culture in a simulated natural environment. Science 296, 1127–1129. doi: 10.1126/science.1070633, PMID: 12004133

[ref11] Kang Q. Sun J. Y. Wang B. W. Sun B. G. (2023). Wine, beer and Chinese *baijiu* in relation to cardiovascular health: the impact of moderate drinking. Food Sci. Human Wellness 12, 1–13. doi: 10.1016/j.fshw.2022.07.013

[ref12] Lei X. J. Zheng J. Zhao D. Qiao Z. W. Fen M. Z. Zhang X. (2022). *Moniliella* aeria sp. nov., a novel yeast isolated from the air of a Wuliangye *baijiu*-making workshop. Int. J. Syst. Evol. Microbiol. 72:005464. doi: 10.1099/ijsem.0.005464 35861490

[ref13] Li X. Chu Q., (2019). The geography of Chinese *baijiu*: Uncovering the spatio-temporal mysteries of high-quality *baijiu* brewing. Xi’an: Northwest University.

[ref14] Li Y. Liu S. Zhang S. Liu T. Qin H. Shen C.. (2022). Spatiotemporal distribution of environmental microbiota in spontaneous fermentation workshop: the case of Chinese *baijiu*. Food Res. Int. 156:111126. doi: 10.1016/j.foodres.2022.111126, PMID: 35651005

[ref15] Louca S. Parfrey L. W. Doebeli M. (2016). Decoupling function and taxonomy in the global ocean microbiome. Science 353, 1272–1277. doi: 10.1126/science.aaf4507, PMID: 27634532

[ref16] Luo J. Zhu S. Wang L. He J. Ouyang L. Zhou J. (2020). Research progress on the composition of brewing microorganisms and flavor substances in strong-flavor *baijiu*. China Brewing 39, 1–6. doi: 10.11882/j.issn.0254-5071.2020.04.001

[ref17] Ma S. Luo H. Zhao D. Qiao Z. Zheng J. An M.. (2022). Environmental factors and interactions among microorganisms drive microbial community succession during fermentation of Nongxiangxing Daqu. Bioresour. Technol. 345:126549. doi: 10.1016/j.biortech.2021.126549, PMID: 34902488

[ref18] Meadow J. F. Altrichter A. E. Kembel S. W. Kline J. Mhuireach K. G. Moriyama M.. (2013). Indoor airborne bacterial communities are influenced by ventilation, occupancy, and outdoor air source. Indoor Air 24, 41–48. doi: 10.1111/ina.12047 23621155 PMC4285785

[ref19] Nguyen N. H. Song Z. Bates S. T. Branco S. Tedersoo L. Menke J.. (2016). FUNGuild: an open annotation tool for parsing fungal community datasets by ecological guild. Fungal Ecol. 20, 241–248. doi: 10.1016/j.funeco.2015.06.006

[ref21] Sakandar H. A. Hussain R. Khan Q. F. Zhang H. (2020). Functional microbiota in Chinese traditional *baijiu* and mijiu Qu (starters): a review. Food Res. Int. 138:109830. doi: 10.1016/j.foodres.2020.109830 33288161

[ref22] Tan Y. Du H. Zhang H. Fang C. Jin G. Chen S.. (2022). Geographically associated fungus-bacterium interactions contribute to the formation of geography-dependent flavor during high-complexity spontaneous fermentation. Microbiol Spectr 10, 1844–1822. doi: 10.1128/spectrum.01844-22 PMC9603688 36135710

[ref23] Tu W. Cao X. Cheng J. Li L. Zhang T. Wu Q.. (2022). Chinese *baijiu*: the perfect works of microorganisms. Front. Microbiol. 13:919044. doi: 10.3389/fmicb.2022.919044, PMID: 35783408 PMC9245514

[ref25] Wang L. (2022). Research trends in jiang-flavor *baijiu* fermentation: from fermentation microecology to environmental ecology. J. Food Sci. 87, 1362–1374. doi: 10.1111/1750-3841.16092 35275413

[ref26] Wang D. Chen L. Yang F. Wang H. Wang L. (2019). Yeasts and their importance to the flavour of traditional Chinese liquor: a review. J. Inst. Brew. 125, 214–221. doi: 10.1002/jib.552

[ref27] Wang P. P. Li Z. Qi T. T. Li X. J. Pan S. Y. (2015). Development of a method for identification and accurate quantitation of aroma compounds in Chinese Daohuaxiang liquors based on SPME using a sol–gel fibre. Food Chem. 169, 230–240. doi: 10.1016/j.foodchem.2014.07.150, PMID: 25236221

[ref28] Wei Y. Zou W. Shen C. H. Yang J. G. (2020). Basic flavor types and component characteristics of Chinese traditional liquors: a review. J. Food Sci. 85, 4096–4107. doi: 10.1111/1750-3841.15536, PMID: 33190291

[ref29] Wu J. J. (2004). Effect of air on Wuliang-flavor Chinese spirit fermentation. Wuxi: Jiangnan University.

[ref31] Xu Y. Sun B. G. Fan G. S. Teng C. Xiong K. Zhu Y. P.. (2017). The brewing process and microbial diversity of strong flavour Chinese spirits: a review. J. Inst. Brew. 123, 5–12. doi: 10.1002/jib.404

[ref32] Xu S. Zhang M. Xu B. Liu L. Sun W. Mu D.. (2022). Microbial communities and flavor formation in the fermentation of Chinese strong-flavor *baijiu* produced from old and new Zaopei. Food Res. Int. 156:111162. doi: 10.1016/j.foodres.2022.111162, PMID: 35651027

[ref33] Xu Y. Zhao J. Liu X. Zhang C. Zhao Z. Li X.. (2022). Flavor mystery of Chinese traditional fermented *baijiu*: the great contribution of ester compounds. Food Chem. 369:130920. doi: 10.1016/j.foodchem.2021.130920, PMID: 34461518

[ref34] Yan Q. Zhang K. Zou W. Hou Y. (2021). Three main flavor types of Chinese *baijiu*: characteristics, research, and perspectives. J. Inst. Brew. 127, 317–326. doi: 10.1002/jib.669

[ref35] Zhai Y. Li X. Wang T. Wang B. Li C. Zeng G. (2018). A review on airborne microorganisms in particulate matters: composition, characteristics, and influence factors. Environ. Int. 113, 74–90. doi: 10.1016/j.envint.2018.01.007, PMID: 29421410

[ref36] Zhang H. Meng Y. Wang Y. Zhou Q. Li A. Liu G.. (2020). Prokaryotic communities in multidimensional bottom-pit-mud from old and young pits used for the production of Chinese strong-flavor *baijiu*. Food Chem. 312:126084. doi: 10.1016/j.foodchem.2019.126084, PMID: 31901820

[ref37] Zheng X. W. Han B. Z. (2016). *Baijiu*, Chinese liquor: history, classification, and manufacture. J. Ethn. Foods 3, 19–25. doi: 10.1016/j.jef.2016.03.001

[ref38] Zhou J. L. Li X. C. Li S. J. Ding H. X. Lang Y. Xu P.. (2024). Airborne microorganisms and key environmental factors shaping their community patterns in the core production area of the Maotai-flavor baijiu. Sci. Total Environ. 912:169010. doi: 10.1016/j.scitotenv.2023.169010, PMID: 38040348

[ref39] Zou W. Zhao C. Luo H. (2018). Diversity and function of microbial community in Chinese strong-flavor *baijiu* ecosystem: a review. Front. Microbiol. 9:671. doi: 10.3389/fmicb.2018.00671, PMID: 29686656 PMC5900010

