# Supplementary material for: Airborne microbes in five important regions of Chinese traditional distilled liquor (Baijiu) brewing: regional and seasonal variations
Source: Front Microbiol. 2024 Jan 9;14:1324722. doi: 10.3389/fmicb.2023.1324722 (PMC10803494; doi:10.3389/fmicb.2023.1324722)
Supplement: Supplementary file 1 [file Data_Sheet_1.pdf]

Supplementary materials for

Airborne microbes in five important regions of Chinese traditional distilled liquor (*Baijiu*) brewing: Regional and seasonal variations

Yan Xu[1], Xue Qiao[2,3,*], Lei He[4], Wujie Wan[4], Zhongjun Xu[2], Xi Shu[2], Cheng Yang[2], Ya Tang[1]

[1]College of Architecture and Environment, Sichuan University, Chengdu 610065, China

[2]Institute of New Energy and Low-carbon Technology, Sichuan University, Chengdu 610065, China

[3]School of Carbon Neutrality Future Technology, Sichuan University, Chengdu 610065, China

[4]College of Life Sciences, Sichuan Normal University, Chengdu 610000, China

[*]Corresponding author, qiao.xue@scu.edu.cn.

The data presented in this study can be obtained from the National Center for Biotechnology Information (NCBI) database (PRJNA1052375 and PRJNA1052644).

Figure captions

Figure S1. Locations of the sampling sites in R1 to R4.

Figure S2. Rarefaction curves of the samples.

Figure S3. Seasonal changes in the top 30 richest genera of (a) fungi and (b) bacteria.

Figure S4. Heatmaps to show the seasonal variations in the trophic modes and growth forms of fungi.

Figure S5. Heatmaps to show the seasonal variations in the predicative functions of bacteria.

Figure S6. The dominant genera contributing to the bacterial and fungal community differences among the regions and between the two seasons.

Table captions

Table S1. Table S1. Information of the 69 sampling sites in the five regions of Sichuan Basin.

Table S2. The total numbers of identified phyla and genera in the ten sample groups.

Table S3. The fungal phyla with relative abundances >0.1% in at least one group.

Table S4. The fungal genera with relative abundances >0.1% in at least one group.

Table S5. The bacterial phyla with relative abundances >0.1% in at least one group.

Table S6. The bacterial genera with relative abundances >0.1% in at least one group.

Table S7. The relative abundances of trophic modes of fungi in each region in summer and winter.

Table S8. The relative abundances of growth forms of fungi in each region in summer and winter.

Table S9. The 30 strongest functions predicted by FAPROTAX in the regions in summer and winter.

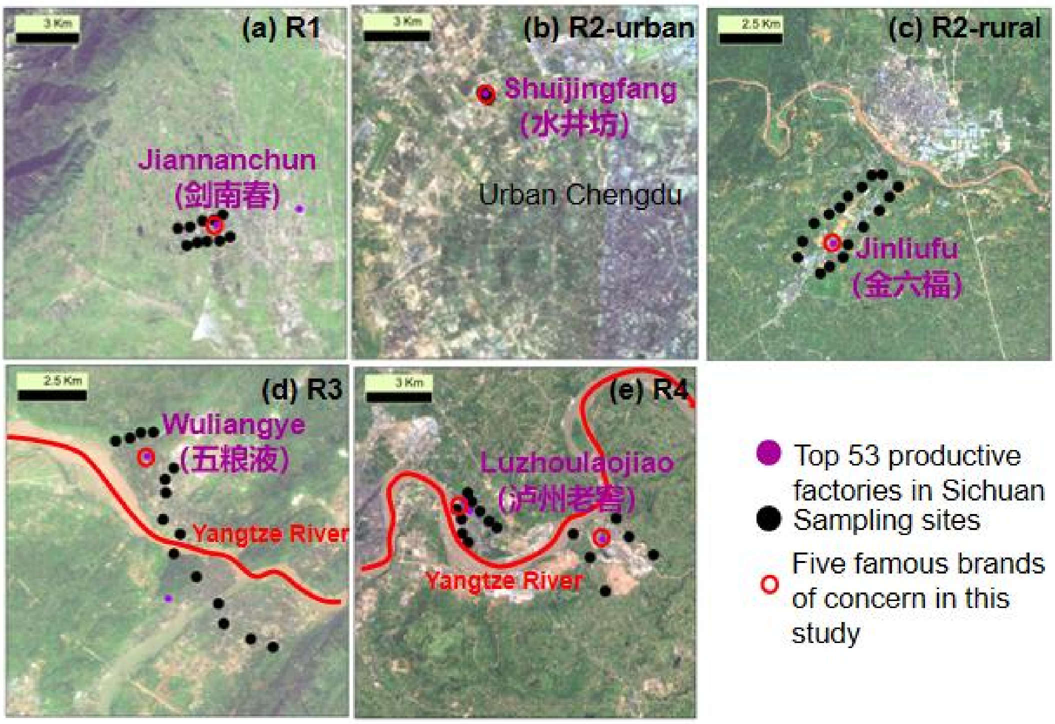

Figure S1. Locations of the sampling sites in R1 to R4.

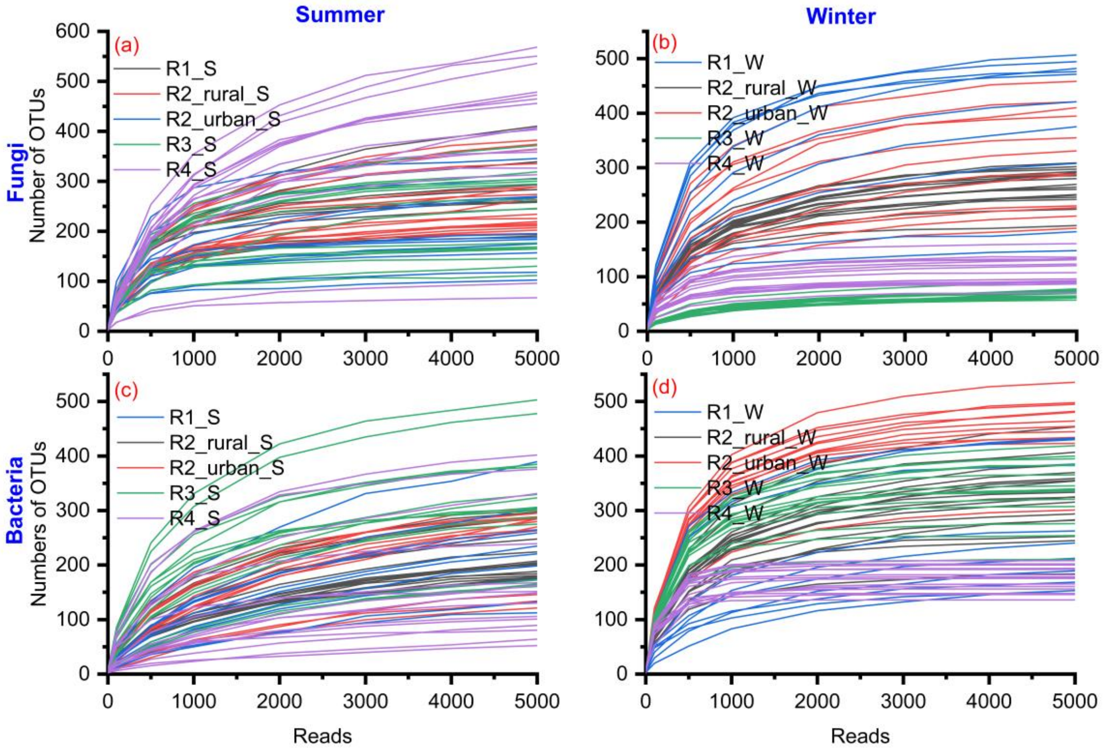

Figure S2. Rarefaction curves of the samples.

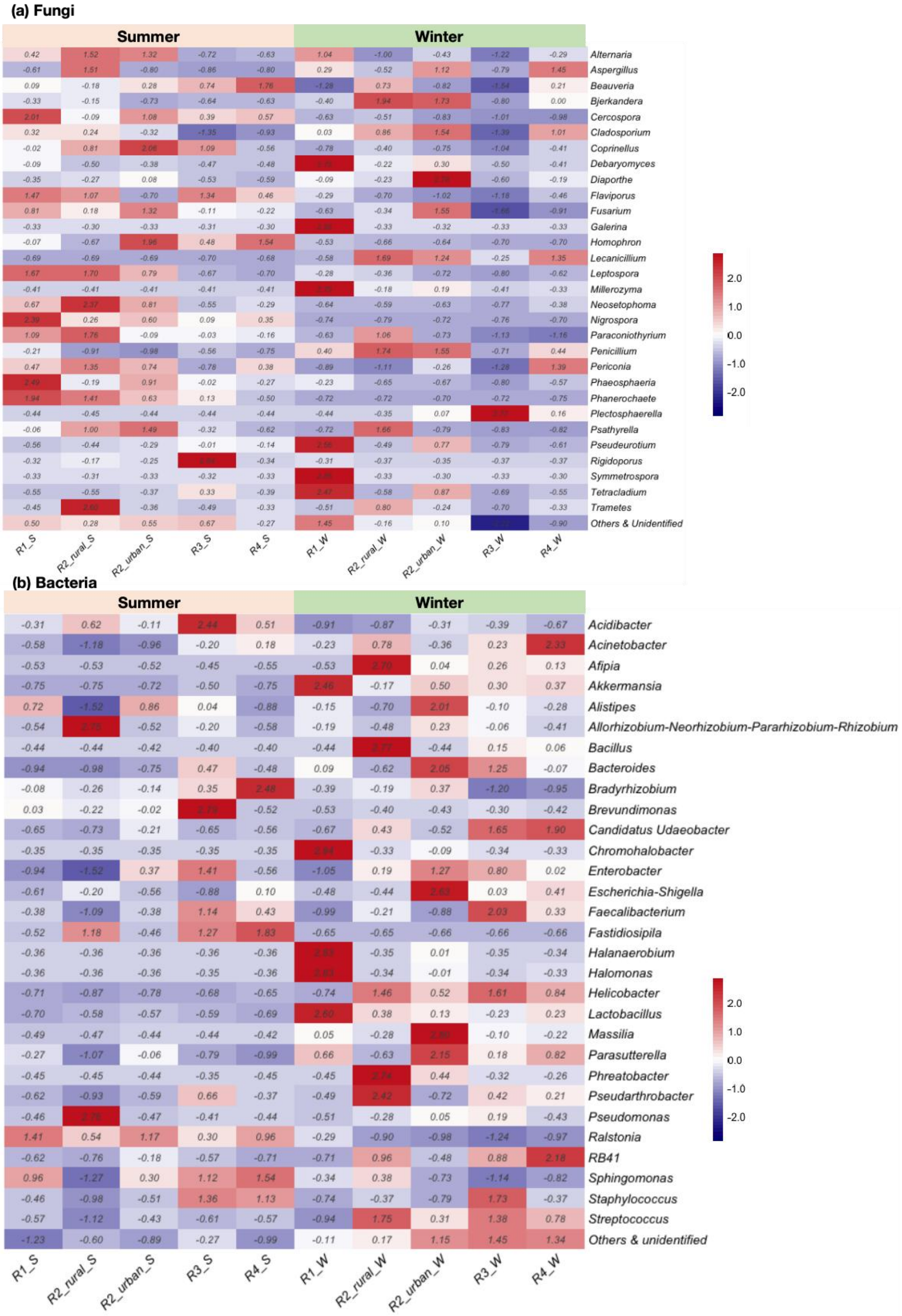

Figure S3. Seasonal changes in the top 30 richest genera of (a) fungi and (b) bacteria.

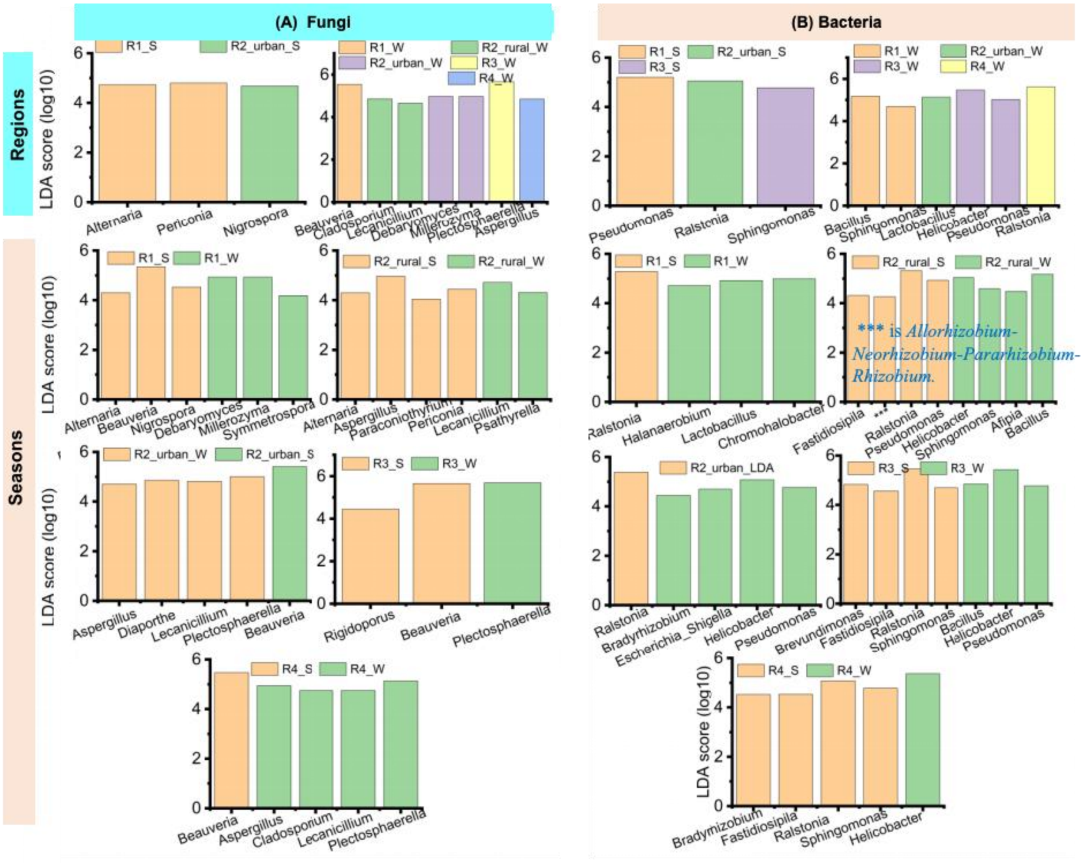

Figure S4. The dominant genera contributing to the bacterial and fungal community differences among the regions and between the two seasons. The comparison was carried out by using the LEfSe analysis for the species with average relative abundance ≥3%. This figure only shows the genus with LDA scores larger than 4.

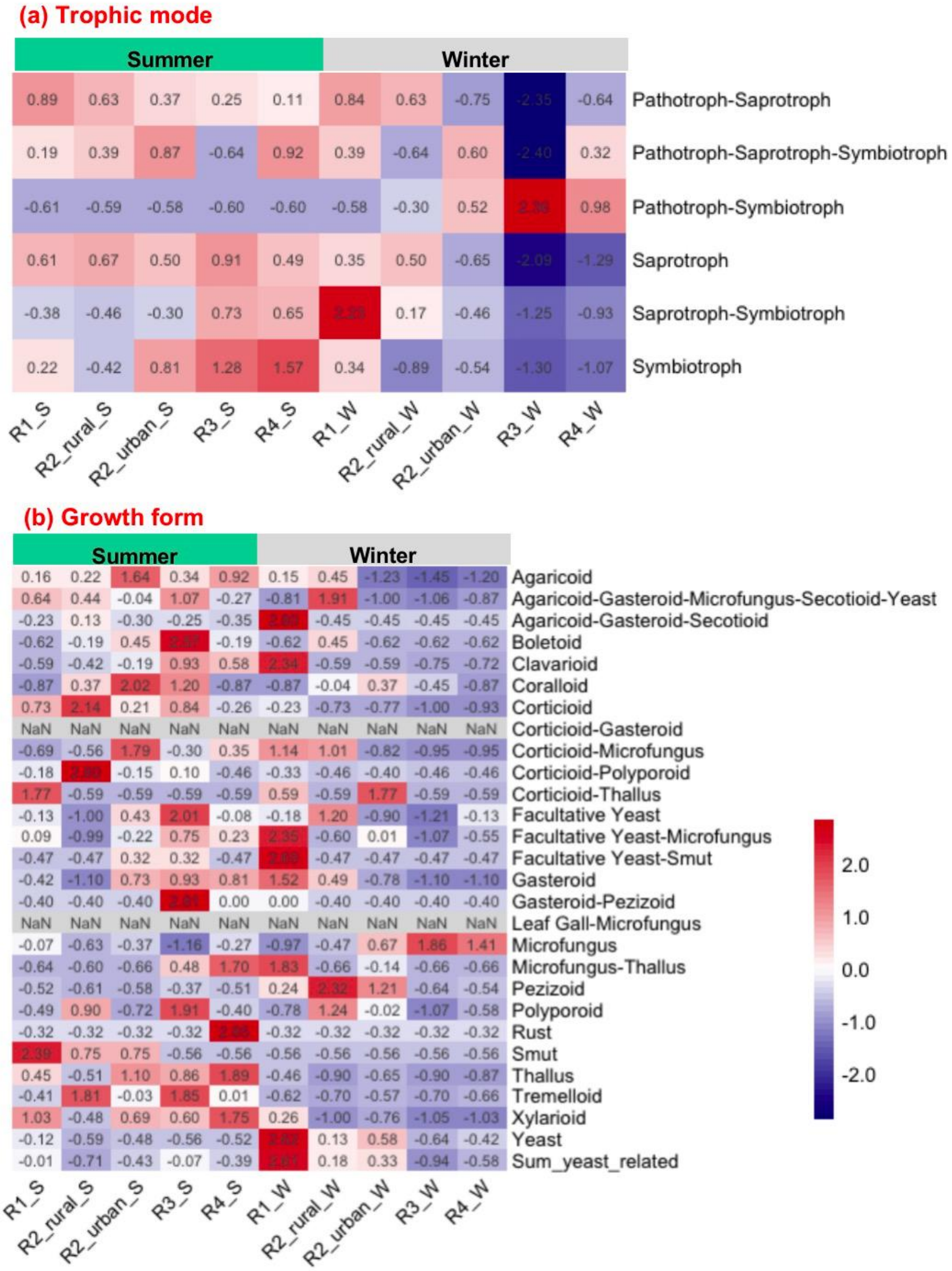

Figure S5. Heatmaps to show the seasonal variations in the trophic modes and growth forms of fungi. Sum_yeast_related is the sum of agaricoid-gasteroid-microfungus-secotioid-yeast, facultatice yeast, facultative yeast-microfungus, facultative yeast-smut, and yeast. NaN suggests the relative abundance of the function was always below 0.001%.

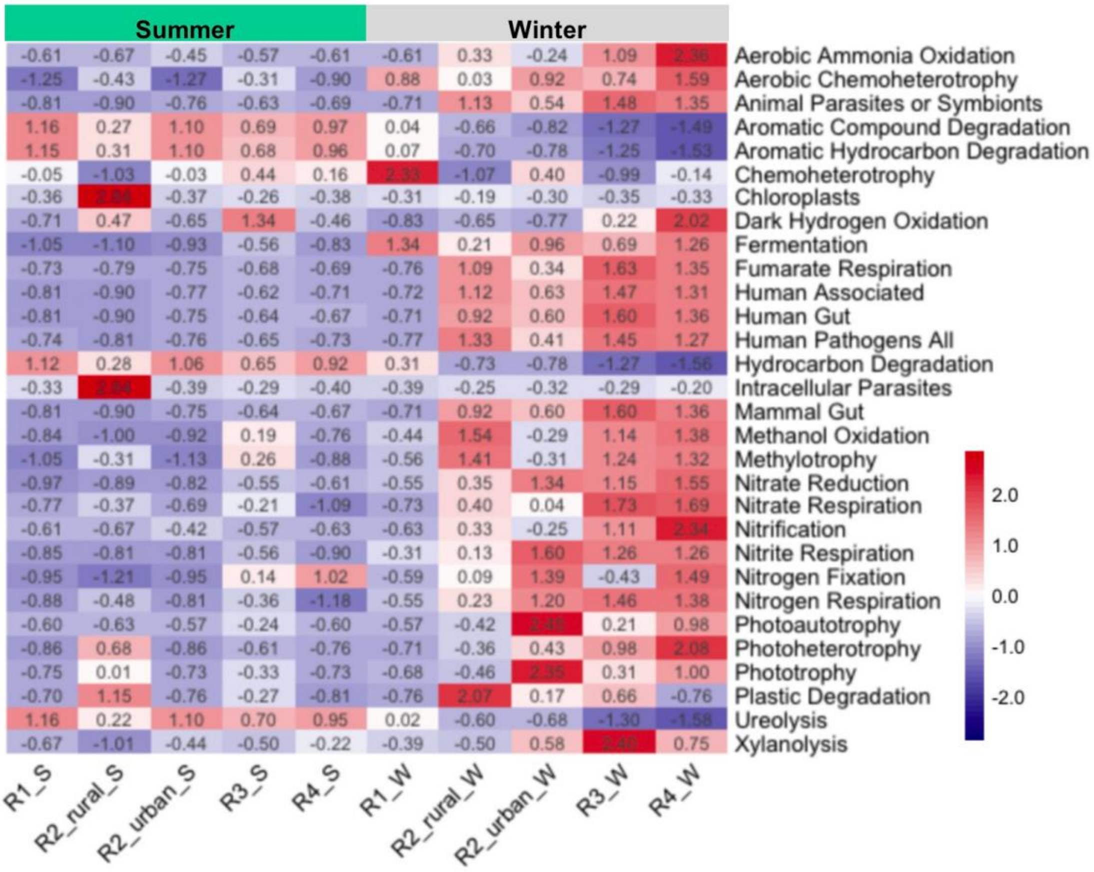

Figure S6. Seasonal variations in the predicative functions of bacteria.

Table S1. Information of the 69 sampling sites in the five regions of Sichuan Basin.

No.	Prefectural City	Famous liquor brand	Longitude (°)	Latitude (°)	Elevation (m a.s.l.)	Summer sample ID	Winter sample ID
R1							
1	Deyang	Jiannanchun	104.165576	31.327314	553	MZ-1-1	MZ-2-1
2	Deyang	Jiannanchun	104.171010	31.329018	560	MZ-1-2	MZ-2-2
3	Deyang	Jiannanchun	104.176036	31.329819	556	MZ-1-3	MZ-2-3
4	Deyang	Jiannanchun	104.182831	31.330376	558	MZ-1-4	MZ-2-4
5	Deyang	Jiannanchun	104.188300	31.332496	546	MZ-1-5	MZ-2-5
6	Deyang	Jiannanchun	104.183649	31.342336	562	MZ-1-6	MZ-2-6
7	Deyang	Jiannanchun	104.179095	31.339699	570	MZ-1-7	MZ-2-7
8	Deyang	Jiannanchun	104.173123	31.338971	560	MZ-1-8	MZ-2-8
9	Deyang	Jiannanchun	104.166612	31.335902	562	MZ-1-9	MZ-2-9
10	Deyang	Jiannanchun	104.160174	31.334865	562	MZ-1-10	MZ-2-10
R2-rural							
11	Chengdu	Jinliufu	103.460995	30.380231	540	QL-1-1	QL-2-1
12	Chengdu	Jinliufu	103.457297	30.376164	542	QL-1-2	QL-2-2
13	Chengdu	Jinliufu	103.453238	30.370690	539	QL-1-3	QL-2-3
14	Chengdu	Jinliufu	103.446737	30.364087	541	QL-1-4	QL-2-4
15	Chengdu	Jinliufu	103.440522	30.357029	549	QL-1-5	QL-2-5
16	Chengdu	Jinliufu	103.438808	30.351951	555	QL-1-6	QL-2-6
17	Chengdu	Jinliufu	103.433188	30.348167	561	QL-1-7	QL-2-7
18	Chengdu	Jinliufu	103.429287	30.345301	560	QL-1-8	QL-2-8
19	Chengdu	Jinliufu	103.453575	30.384447	516	QL-1-9	QL-2-9
20	Chengdu	Jinliufu	103.448993	30.383882	513	QL-1-10	QL-2-10
21	Chengdu	Jinliufu	103.445002	30.379166	522	QL-1-11	QL-2-11
22	Chengdu	Jinliufu	103.439136	30.375338	521	QL-1-12	QL-2-12
23	Chengdu	Jinliufu	103.436025	30.370856	524	QL-1-13	QL-2-13
24	Chengdu	Jinliufu	103.431414	30.367981	527	QL-1-14	QL-2-14
25	Chengdu	Jinliufu	103.425105	30.364194	534	QL-1-15	QL-2-15
26	Chengdu	Jinliufu	103.420531	30.357020	548	QL-1-16	QL-2-16
27	Chengdu	Jinliufu	103.418798	30.350846	560	QL-1-17	QL-2-17
R2-urban							
28	Chengdu	Shuijinfang	103.996948	30.730519	477	JN-1-1	JN-2-1
29	Chengdu	Shuijinfang	103.999288	30.728510	473	JN-1-2	JN-2-2
30	Chengdu	Shuijinfang	104.000277	30.727692	477	JN-1-3	JN-2-3
31	Chengdu	Shuijinfang	103.996948	30.730519	477	JN-1-4	JN-2-4
32	Chengdu	Shuijinfang	103.999288	30.728510	473	JN-1-5	JN-2-5
33	Chengdu	Shuijinfang	104.000277	30.727692	477	JN-1-6	JN-2-6
34	Chengdu	Shuijinfang	103.997534	30.731283	469	JN-1-7	JN-2-7
35	Chengdu	Shuijinfang	104.000073	30.729319	470	JN-1-8	JN-2-8
36	Chengdu	Shuijinfang	104.000976	30.728386	464	JN-1-9	JN-2-9
37	Chengdu	Shuijinfang	103.997534	30.731283	469	JN-1-10	JN-2-10
38	Chengdu	Shuijinfang	104.000073	30.729319	470	JN-1-11	JN-2-11
39	Chengdu	Shuijinfang	104.000976	30.728386	464	JN-1-12	JN-2-12
R3							
40	Yibin	Wuliangye	104.644946	28.747131	267	YB-1-1	YB-2-1
41	Yibin	Wuliangye	104.636026	28.749541	264	YB-1-2	YB-2-2
42	Yibin	Wuliangye	104.625060	28.754346	303	YB-1-3	YB-2-3
43	Yibin	Wuliangye	104.622465	28.761177	231	YB-1-4	YB-2-4
44	Yibin	Wuliangye	104.613246	28.770057	237	YB-1-5	YB-2-5

No .	Prefectural City	Famous liquor brand	Longitude (°)	Latitude (°)	Elevation (m a.s.l.)	Summer sample ID	Winter sample ID
45	Yibin	Wuliangye	104.603844	28.777620	252	YB-1-6	YB-2-6
46	Yibin	Wuliangye	104.605951	28.784466	262	YB-1-7	YB-2-7
47	Yibin	Wuliangye	104.598829	28.789223	287	YB-1-8	YB-2-8
48	Yibin	Wuliangye	104.599388	28.798604	299	YB-1-9	YB-2-9
49	Yibin	Wuliangye	104.598796	28.803152	306	YB-1-10	YB-2-10
50	Yibin	Wuliangye	104.602010	28.807282	296	YB-1-11	YB-2-11
51	Yibin	Wuliangye	104.593156	28.819260	312	YB-1-12	YB-2-12
52	Yibin	Wuliangye	104.588228	28.818853	332	YB-1-13	YB-2-13
53	Yibin	Wuliangye	104.584023	28.816837	291	YB-1-14	YB-2-14
54	Yibin	Wuliangye	104.578492	28.815530	281	YB-1-15	YB-2-15
R4							
55	Luzhou	Luzhoulaojiao	105.546601	28.888766	187	LZ-1-1	LZ-2-1
56	Luzhou	Luzhoulaojiao	105.555510	28.878890	232	LZ-1-2	LZ-2-2
57	Luzhou	Luzhoulaojiao	105.563845	28.864528	260	LZ-1-3	LZ-2-3
58	Luzhou	Luzhoulaojiao	105.567670	28.896450	204	LZ-1-4	LZ-2-4
59	Luzhou	Luzhoulaojiao	105.574453	28.888656	237	LZ-1-5	LZ-2-5
60	Luzhou	Luzhoulaojiao	105.587104	28.881295	233	LZ-1-6	LZ-2-6
61	Luzhou	Luzhoulaojiao	105.492937	28.904752	247	LZ-1-7	LZ-2-7
62	Luzhou	Luzhoulaojiao	105.495090	28.900970	221	LZ-1-8	LZ-2-8
63	Luzhou	Luzhoulaojiao	105.499553	28.896821	256	LZ-1-9	LZ-2-9
64	Luzhou	Luzhoulaojiao	105.504235	28.892716	254	LZ-1-10	LZ-2-10
65	Luzhou	Luzhoulaojiao	105.508366	28.890019	257	LZ-1-11	LZ-2-11
66	Luzhou	Luzhoulaojiao	105.487606	28.897240	195	LZ-1-12	LZ-2-12
67	Luzhou	Luzhoulaojiao	105.490424	28.892980	193	LZ-1-13	LZ-2-13
68	Luzhou	Luzhoulaojiao	105.491076	28.886742	198	LZ-1-14	LZ-2-14
69	Luzhou	Luzhoulaojiao	105.494172	28.883058	221	LZ-1-15	LZ-2-15

Table S2. The total numbers of identified phyla and genera in the ten sample groups.

Region	Season	Group_ID	Fungi		Bacteria	
			Phyla	Genera	Phyla	Genera
R1	Summer	R1_S	9	344	36	453
	Winter	R1_W	8	406	34	504
R2_rural	Summer	R2_rural_S	5	348	35	411
	Winter	R2_rural_W	8	257	42	674
R2_urban	Summer	R2_urban_S	8	314	36	583
	Winter	R2_urban_W	9	419	42	665
R3	Summer	R3_S	8	391	37	632
	Winter	R3_W	5	324	45	804
R4	Summer	R4_S	7	145	33	467
	Winter	R4_W	7	242	43	820

Table S3. The fungal phyla with relative abundances >0.1% in at least one group.

**Phyla**	**Relative abundance (%)**												
	**Mean**	**Min**	**Max**	**R1_S**	**R1_W**	**R2_rural_S**	**R2_rural_W**	**R2_urban_S**	**R2_urban_W**	**R3_S**	**R3_W**	**R4_S**	**R4_W**
Ascomycota	83.46	73.15	99.18	79.85	75.72	76.90	84.03	76.25	90.55	73.15	99.18	84.38	94.61
Basidiomycota	12.88	0.71	21.00	15.91	16.57	20.85	13.32	18.40	6.68	21.00	0.71	11.35	4.01
Chytridiomycota	0.09	0.00	0.38	0.00	0.38	0.00	0.36	0.03	0.04	0.06	0.00	0.06	0.00
Glomeromycota	0.10	0.00	0.52	0.11	0.52	0.00	0.04	0.07	0.06	0.17	0.00	0.06	0.00
GS01	0.07	0.00	0.18	0.00	0.11	0.01	0.02	0.15	0.02	0.18	0.00	0.10	0.08
Mortierellomycota	0.23	0.00	1.16	0.13	1.16	0.05	0.20	0.16	0.26	0.17	0.00	0.13	0.06
Mucoromycota	0.08	0.01	0.26	0.14	0.05	0.26	0.12	0.07	0.04	0.06	0.01	0.01	0.04
Unidentified	3.07	0.10	5.48	3.84	5.48	1.93	1.92	4.82	2.35	5.18	0.10	3.91	1.20

Table S4. The fungal genera with relative abundances >0.1% in at least one group.

No.	Genera	Relative abundance (%)												
		Mean	Min	Max	R1_S	R1_W	R2_rural_S	R2_rural_W	R2_urban_S	R2_urban_W	R3_S	R4_S	R3_W	R4_W
1	*Acremonium*	0.14	0.00	0.53	0.06	0.53	0.01	0.15	0.02	0.53	0.04	0.01	0.00	0.05
2	*Acrocalymma*	0.04	0.00	0.21	0.21	0.00	0.07	0.00	0.08	0.02	0.04	0.02	0.00	0.00
3	*Agaricus*	0.03	0.00	0.14	0.03	0.01	0.14	0.00	0.03	0.00	0.04	0.01	0.00	0.00
4	*Alternaria*	1.22	0.06	2.66	1.62	2.20	2.66	0.27	2.47	0.81	0.54	0.62	0.06	0.94
5	*Apiotrichum*	0.15	0.00	0.51	0.21	0.02	0.00	0.00	0.39	0.00	0.51	0.31	0.01	0.02
6	*Arthothelium*	0.02	0.00	0.11	0.00	0.01	0.00	0.00	0.01	0.02	0.02	0.11	0.00	0.00
7	*Arthrinium*	0.10	0.02	0.21	0.08	0.09	0.05	0.11	0.05	0.07	0.13	0.21	0.02	0.21
8	*Ascobolus*	0.19	0.00	0.66	0.13	0.66	0.00	0.12	0.24	0.17	0.24	0.09	0.02	0.22
9	*Aspergillus*	5.02	1.49	11.21	2.53	6.22	11.21	2.87	1.76	9.64	1.49	1.75	1.79	10.98
10	*Aureobasidium*	0.02	0.00	0.12	0.02	0.00	0.00	0.12	0.00	0.00	0.02	0.01	0.00	0.02
11	*Auricularia*	0.03	0.00	0.18	0.00	0.00	0.08	0.00	0.18	0.00	0.05	0.00	0.00	0.00
12	*Bacidina*	0.16	0.00	0.81	0.42	0.02	0.00	0.00	0.81	0.02	0.22	0.15	0.00	0.01
13	*Bartalinia*	0.03	0.00	0.10	0.01	0.02	0.03	0.00	0.05	0.02	0.10	0.04	0.00	0.01
14	*Beauveria*	27.38	3.39	54.79	28.80	7.46	24.54	38.76	31.77	14.61	38.94	54.79	3.39	30.70
15	*Beltrania*	0.02	0.00	0.14	0.00	0.14	0.00	0.00	0.00	0.01	0.01	0.00	0.00	0.00
16	*Bipolaris*	0.10	0.00	0.42	0.02	0.07	0.42	0.09	0.28	0.01	0.01	0.06	0.00	0.01
17	*Bjerkandera*	0.82	0.04	2.69	0.50	0.43	0.67	2.69	0.11	2.49	0.20	0.21	0.04	0.82
18	*Blakeslea*	0.04	0.00	0.25	0.13	0.00	0.25	0.00	0.03	0.00	0.02	0.00	0.00	0.00
19	*Botryosphaeria*	0.05	0.00	0.39	0.03	0.00	0.05	0.00	0.39	0.01	0.00	0.01	0.00	0.04
20	*Byssochlamys*	0.07	0.00	0.23	0.02	0.10	0.06	0.20	0.01	0.06	0.00	0.02	0.02	0.23
21	*Calonectria*	0.09	0.00	0.53	0.00	0.53	0.11	0.12	0.00	0.12	0.01	0.00	0.00	0.05
22	*Candida*	0.04	0.00	0.19	0.02	0.02	0.01	0.19	0.00	0.11	0.00	0.00	0.00	0.03
23	*Capronia*	0.04	0.00	0.16	0.04	0.00	0.01	0.00	0.08	0.00	0.16	0.03	0.00	0.03
24	*Ceratocystis*	0.14	0.00	1.03	0.00	0.09	0.00	0.05	0.00	1.03	0.00	0.00	0.00	0.26
25	*Cercospora*	0.60	0.00	1.79	1.79	0.23	0.55	0.30	1.24	0.11	0.83	0.94	0.00	0.02
26	*Chaetomium*	0.08	0.00	0.45	0.04	0.09	0.00	0.05	0.03	0.45	0.07	0.04	0.01	0.06
27	*Chaetosphaeria*	0.07	0.00	0.41	0.00	0.07	0.05	0.04	0.01	0.41	0.04	0.00	0.00	0.02
28	*Chaetothyrina*	0.07	0.00	0.36	0.00	0.00	0.36	0.22	0.00	0.08	0.00	0.00	0.00	0.01
29	*Chalara*	0.09	0.00	0.47	0.00	0.47	0.01	0.09	0.00	0.25	0.02	0.02	0.00	0.02
30	*Cheilymenia*	0.18	0.00	0.68	0.02	0.29	0.00	0.67	0.02	0.68	0.07	0.02	0.00	0.03
31	*Cistella*	0.09	0.00	0.44	0.03	0.44	0.00	0.09	0.06	0.14	0.07	0.01	0.00	0.01
32	*Cladosporium*	5.59	2.04	9.52	6.40	5.66	6.19	7.79	4.76	9.52	2.14	3.20	2.04	8.18
33	*Claroideoglomus*	0.03	0.00	0.24	0.00	0.24	0.00	0.00	0.00	0.01	0.00	0.01	0.00	0.00
34	*Clavaria*	0.05	0.00	0.27	0.00	0.27	0.00	0.01	0.02	0.01	0.08	0.07	0.00	0.00
35	*Claviceps*	0.13	0.00	0.52	0.52	0.22	0.00	0.35	0.00	0.12	0.02	0.08	0.02	0.01
36	*Clitopilus*	0.04	0.00	0.11	0.02	0.11	0.08	0.11	0.01	0.01	0.03	0.00	0.00	0.00
37	*Clonostachys*	0.06	0.00	0.17	0.08	0.04	0.06	0.02	0.05	0.09	0.17	0.03	0.00	0.05
38	*Colletotrichum*	0.15	0.01	0.79	0.08	0.01	0.79	0.13	0.19	0.14	0.04	0.10	0.01	0.02
39	*Coniochaeta*	0.04	0.00	0.11	0.04	0.11	0.00	0.03	0.03	0.07	0.02	0.04	0.00	0.04

No.	Genera	Relative abundance (%)												
		Mean	Min	Max	R1_S	R1_W	R2_rural_S	R2_rural_W	R2_urban_S	R2_urban_W	R3_S	R4_S	R3_W	R4_W
40	*Coniothyrium*	0.05	0.00	0.20	0.00	0.00	0.00	0.01	0.13	0.01	0.20	0.04	0.00	0.06
41	*Coprinellus*	0.70	0.07	1.95	0.69	0.23	1.19	0.46	1.95	0.25	1.36	0.36	0.07	0.45
42	*Coprinopsis*	0.14	0.00	0.61	0.61	0.20	0.06	0.13	0.14	0.03	0.19	0.04	0.00	0.05
43	*Corynespora*	0.03	0.00	0.24	0.03	0.00	0.02	0.00	0.02	0.00	0.02	0.24	0.00	0.00
44	*Cosmospora*	0.01	0.00	0.11	0.00	0.11	0.00	0.00	0.00	0.00	0.00	0.00	0.00	0.00
45	*Cryptocoryneum*	0.02	0.00	0.14	0.01	0.00	0.01	0.00	0.14	0.03	0.01	0.01	0.00	0.03
46	*Curvularia*	0.14	0.00	0.69	0.07	0.07	0.69	0.02	0.06	0.04	0.07	0.33	0.00	0.06
47	*Cyphellophora*	0.16	0.02	0.46	0.46	0.12	0.02	0.10	0.23	0.12	0.21	0.07	0.12	0.21
48	*Dactylonectria*	0.30	0.00	1.08	0.20	1.08	0.00	0.29	0.08	0.58	0.36	0.20	0.10	0.10
49	*Daldinia*	0.04	0.00	0.23	0.02	0.00	0.03	0.00	0.23	0.00	0.09	0.03	0.00	0.02
50	*Darksidea*	0.07	0.00	0.50	0.03	0.50	0.00	0.00	0.00	0.17	0.02	0.00	0.00	0.00
51	*Debaryomyces*	1.08	0.00	6.96	0.88	6.96	0.00	0.61	0.26	1.72	0.06	0.05	0.00	0.20
52	*Devriesia*	0.02	0.00	0.10	0.00	0.00	0.07	0.00	0.00	0.10	0.00	0.00	0.00	0.01
53	*Diaporthe*	0.44	0.01	2.44	0.19	0.38	0.25	0.28	0.50	2.44	0.06	0.02	0.01	0.31
54	*Didymella*	0.05	0.00	0.25	0.07	0.02	0.25	0.00	0.01	0.02	0.09	0.04	0.00	0.03
55	*Didymocrea*	0.11	0.00	0.89	0.04	0.04	0.02	0.01	0.06	0.00	0.02	0.01	0.01	0.89
56	*Dissoconium*	0.04	0.00	0.14	0.06	0.00	0.14	0.01	0.02	0.04	0.06	0.06	0.00	0.00
57	*Dominikia*	0.01	0.00	0.11	0.00	0.11	0.00	0.00	0.00	0.01	0.02	0.00	0.00	0.00
58	*Duportella*	0.07	0.00	0.29	0.02	0.00	0.18	0.00	0.29	0.01	0.22	0.03	0.00	0.00
59	*Eichleriella*	0.05	0.00	0.17	0.10	0.00	0.10	0.01	0.17	0.00	0.07	0.02	0.00	0.00
60	*Emericellopsis*	0.02	0.00	0.14	0.01	0.01	0.00	0.01	0.00	0.14	0.00	0.00	0.00	0.00
61	*Exidia*	0.05	0.00	0.21	0.02	0.01	0.21	0.00	0.05	0.01	0.17	0.03	0.00	0.00
62	*Exophiala*	0.08	0.00	0.20	0.09	0.20	0.00	0.04	0.15	0.09	0.07	0.09	0.00	0.10
63	*Exserohilum*	0.02	0.00	0.19	0.19	0.00	0.00	0.00	0.00	0.01	0.00	0.00	0.00	0.00
64	*Fellomyces*	0.02	0.00	0.11	0.11	0.00	0.00	0.00	0.04	0.00	0.03	0.00	0.00	0.05
65	*Fellozyma*	0.02	0.00	0.20	0.00	0.20	0.00	0.00	0.00	0.00	0.01	0.00	0.00	0.00
66	*Flaviporus*	0.87	0.03	1.91	1.91	0.66	1.63	0.37	0.37	0.14	1.82	1.19	0.03	0.54
67	*Flavodon*	0.28	0.00	1.01	0.22	0.00	0.49	0.73	1.01	0.01	0.19	0.08	0.00	0.01
68	*Fusarium*	0.31	0.02	0.58	0.45	0.20	0.34	0.25	0.54	0.58	0.29	0.27	0.02	0.15
69	*Fusicladium*	0.02	0.00	0.14	0.00	0.14	0.07	0.00	0.00	0.00	0.00	0.00	0.00	0.00
70	*Fusicolla*	0.14	0.01	0.33	0.03	0.33	0.07	0.06	0.16	0.26	0.28	0.16	0.01	0.05
71	*Fusidium*	0.04	0.00	0.17	0.07	0.17	0.00	0.01	0.02	0.03	0.02	0.07	0.00	0.01
72	*Gaeumannomyces*	0.02	0.00	0.11	0.11	0.04	0.02	0.00	0.00	0.02	0.00	0.00	0.00	0.00
73	*Galerina*	0.35	0.00	3.43	0.00	3.43	0.03	0.00	0.00	0.01	0.02	0.03	0.00	0.00
74	*Ganoderma*	0.08	0.00	0.34	0.08	0.01	0.34	0.00	0.14	0.00	0.10	0.10	0.00	0.00
75	*Geastrum*	0.07	0.00	0.23	0.00	0.23	0.00	0.10	0.13	0.02	0.12	0.08	0.00	0.00
76	*Geoglossum*	0.05	0.00	0.23	0.04	0.02	0.00	0.00	0.03	0.00	0.23	0.20	0.00	0.00
77	*Geosmithia*	0.04	0.00	0.13	0.00	0.03	0.00	0.13	0.04	0.05	0.00	0.00	0.01	0.12
78	*Gibberella*	0.22	0.00	0.74	0.50	0.09	0.36	0.01	0.26	0.11	0.02	0.00	0.07	0.74
79	*Gymnopilus*	0.04	0.00	0.13	0.08	0.00	0.13	0.00	0.04	0.00	0.06	0.04	0.00	0.00
80	*Gyoerffyella*	0.04	0.00	0.12	0.04	0.01	0.12	0.00	0.03	0.04	0.09	0.07	0.00	0.00
81	*Hanseniaspora*	0.03	0.00	0.11	0.05	0.05	0.00	0.00	0.02	0.03	0.06	0.11	0.00	0.00

No.	Genera	Relative abundance (%)												
		Mean	Min	Max	R1_S	R1_W	R2_rural_S	R2_rural_W	R2_urban_S	R2_urban_W	R3_S	R4_S	R3_W	R4_W
82	*Hansfordia*	0.05	0.00	0.15	0.03	0.01	0.13	0.15	0.13	0.05	0.01	0.01	0.00	0.00
83	*Hemimycena*	0.02	0.00	0.13	0.02	0.02	0.01	0.00	0.02	0.00	0.13	0.02	0.00	0.00
84	*Homophron*	0.79	0.01	2.97	0.71	0.20	0.04	0.05	2.97	0.07	1.32	2.51	0.01	0.01
85	*Humicola*	0.12	0.00	0.49	0.05	0.49	0.00	0.02	0.07	0.23	0.21	0.11	0.01	0.02
86	*Hymenochaete*	0.02	0.00	0.11	0.02	0.03	0.11	0.00	0.01	0.00	0.04	0.00	0.00	0.00
87	*Hyphoderma*	0.02	0.00	0.10	0.01	0.00	0.04	0.00	0.01	0.00	0.10	0.04	0.00	0.00
88	*Hyphodontia*	0.08	0.00	0.39	0.00	0.00	0.39	0.00	0.09	0.01	0.20	0.06	0.00	0.00
89	*Hypholoma*	0.02	0.00	0.12	0.00	0.12	0.01	0.06	0.01	0.01	0.01	0.00	0.00	0.01
90	*Hypoxylon*	0.08	0.00	0.31	0.07	0.31	0.04	0.00	0.08	0.06	0.12	0.17	0.00	0.00
91	*Idriella*	0.02	0.00	0.15	0.00	0.15	0.00	0.01	0.00	0.01	0.00	0.00	0.00	0.00
92	*Ilyonectria*	0.06	0.00	0.19	0.00	0.16	0.02	0.05	0.04	0.19	0.09	0.03	0.00	0.00
93	*Kabatiella*	0.05	0.00	0.20	0.00	0.07	0.03	0.02	0.00	0.11	0.04	0.00	0.02	0.20
94	*Kazachstania*	0.02	0.00	0.13	0.00	0.13	0.00	0.00	0.00	0.01	0.00	0.00	0.00	0.02
95	*Knufia*	0.11	0.00	0.24	0.14	0.24	0.11	0.00	0.17	0.22	0.14	0.01	0.00	0.03
96	*Lasiodiplodia*	0.02	0.00	0.17	0.00	0.01	0.01	0.00	0.01	0.17	0.01	0.01	0.00	0.02
97	*Lecanicillium*	1.79	0.02	6.11	0.03	0.31	0.03	6.11	0.04	4.94	0.02	0.05	1.15	5.23
98	*Lentinus*	0.05	0.00	0.45	0.01	0.00	0.45	0.00	0.00	0.00	0.00	0.00	0.00	0.00
99	*Leohumicola*	0.19	0.00	0.51	0.18	0.45	0.13	0.07	0.02	0.51	0.35	0.15	0.00	0.04
100	*Lepiota*	0.09	0.00	0.86	0.01	0.86	0.00	0.00	0.00	0.00	0.00	0.00	0.00	0.00
101	*Lepista*	0.02	0.00	0.14	0.14	0.00	0.01	0.02	0.01	0.00	0.00	0.00	0.00	0.00
102	*Leptospora*	0.31	0.00	0.96	0.95	0.20	0.96	0.17	0.61	0.03	0.05	0.04	0.00	0.07
103	*Lindgomyces*	0.06	0.00	0.26	0.00	0.26	0.03	0.09	0.01	0.07	0.06	0.04	0.00	0.00
104	*Lyomyces*	0.08	0.00	0.24	0.16	0.02	0.24	0.01	0.10	0.00	0.19	0.05	0.00	0.00
105	*Malassezia*	0.26	0.01	1.42	0.13	0.11	0.01	1.42	0.05	0.04	0.08	0.46	0.15	0.17
106	*Meira*	0.06	0.00	0.26	0.21	0.01	0.00	0.00	0.26	0.00	0.09	0.03	0.00	0.00
107	*Metarhizium*	0.06	0.00	0.29	0.03	0.06	0.00	0.29	0.10	0.08	0.01	0.01	0.00	0.02
108	*Microascus*	0.03	0.00	0.13	0.00	0.00	0.01	0.13	0.12	0.02	0.00	0.01	0.00	0.05
109	*Millerozyma*	0.72	0.00	5.61	0.00	5.61	0.00	0.41	0.00	1.05	0.00	0.00	0.00	0.15
110	*Minutisphaera*	0.06	0.00	0.38	0.02	0.38	0.00	0.02	0.05	0.08	0.01	0.00	0.00	0.01
111	*Monocillium*	0.07	0.00	0.65	0.01	0.01	0.00	0.00	0.00	0.00	0.65	0.02	0.00	0.00
112	*Monographella*	0.08	0.00	0.53	0.03	0.53	0.00	0.00	0.05	0.12	0.00	0.02	0.00	0.01
113	*Mortierella*	0.16	0.00	0.57	0.12	0.57	0.05	0.18	0.15	0.22	0.13	0.12	0.00	0.04
114	*Mrakia*	0.05	0.00	0.36	0.00	0.02	0.00	0.36	0.00	0.02	0.01	0.01	0.01	0.07
115	*Mycoacia*	0.09	0.00	0.76	0.02	0.76	0.04	0.00	0.00	0.03	0.02	0.01	0.00	0.00
116	*Mycodiella*	0.06	0.00	0.37	0.00	0.12	0.37	0.00	0.00	0.09	0.00	0.00	0.00	0.00
117	*Mycoleptodiscus*	0.07	0.00	0.23	0.04	0.23	0.01	0.02	0.14	0.02	0.15	0.03	0.00	0.08
118	*Mycosphaerelloides*	0.04	0.00	0.22	0.00	0.22	0.05	0.07	0.00	0.05	0.00	0.00	0.00	0.01
119	*Myrmecridium*	0.04	0.00	0.26	0.01	0.01	0.01	0.00	0.01	0.03	0.02	0.26	0.00	0.01
120	*Myrothecium*	0.02	0.00	0.10	0.10	0.00	0.02	0.00	0.06	0.01	0.01	0.00	0.00	0.00
121	*Naganishia*	0.02	0.00	0.12	0.01	0.12	0.01	0.01	0.00	0.01	0.00	0.01	0.00	0.04
122	*Neodevriesia*	0.05	0.00	0.10	0.00	0.02	0.07	0.04	0.03	0.05	0.08	0.08	0.02	0.10
123	*Neofusicoccum*	0.03	0.00	0.25	0.05	0.00	0.02	0.00	0.25	0.00	0.00	0.01	0.00	0.00

No.	Genera	Relative abundance (%)												
		Mean	Min	Max	R1_S	R1_W	R2_rural_S	R2_rural_W	R2_urban_S	R2_urban_W	R3_S	R4_S	R3_W	R4_W
124	*Neonectria*	0.07	0.00	0.17	0.03	0.17	0.09	0.07	0.06	0.16	0.09	0.04	0.00	0.01
125	*Neosetophoma*	0.56	0.01	2.25	1.04	0.10	2.25	0.14	1.14	0.11	0.17	0.35	0.01	0.29
126	*Niesslia*	0.03	0.00	0.25	0.00	0.00	0.00	0.25	0.00	0.02	0.00	0.00	0.00	0.00
127	*Nigrograna*	0.07	0.00	0.49	0.01	0.11	0.04	0.49	0.00	0.03	0.05	0.00	0.00	0.00
128	*Nigrospora*	0.81	0.00	3.25	3.25	0.05	1.07	0.00	1.42	0.07	0.90	1.16	0.03	0.09
129	*Ochroconis*	0.21	0.00	0.44	0.41	0.09	0.37	0.11	0.29	0.09	0.25	0.44	0.01	0.00
130	*Ophiosphaerella*	0.10	0.00	0.19	0.14	0.19	0.13	0.02	0.07	0.10	0.13	0.18	0.09	0.00
131	*Oxyporus*	0.04	0.00	0.26	0.00	0.01	0.26	0.01	0.05	0.01	0.00	0.00	0.01	0.01
132	*Papiliotrema*	0.12	0.00	0.66	0.17	0.05	0.07	0.66	0.02	0.02	0.05	0.09	0.00	0.09
133	*Paraconiothyrium*	0.72	0.02	1.77	1.37	0.34	1.77	1.35	0.66	0.28	0.70	0.62	0.04	0.02
134	*Paracremonium*	0.05	0.00	0.15	0.00	0.15	0.12	0.00	0.10	0.13	0.00	0.03	0.00	0.00
135	*Paraphaeosphaeria*	0.03	0.00	0.11	0.02	0.01	0.02	0.01	0.11	0.02	0.05	0.01	0.00	0.00
136	*Penicillium*	0.37	0.11	0.82	0.31	0.47	0.13	0.82	0.11	0.77	0.22	0.17	0.18	0.48
137	*Peniophora*	0.13	0.00	0.35	0.35	0.01	0.33	0.04	0.30	0.02	0.18	0.08	0.00	0.00
138	*Perenniporia*	0.08	0.00	0.56	0.02	0.01	0.56	0.01	0.02	0.00	0.13	0.08	0.00	0.00
139	*Periconia*	1.59	0.10	3.21	2.14	0.55	3.16	0.30	2.46	1.29	0.68	2.03	0.10	3.21
140	*Peroneutypa*	0.05	0.00	0.36	0.04	0.04	0.36	0.00	0.01	0.00	0.01	0.00	0.00	0.00
141	*Pestalotiopsis*	0.05	0.00	0.15	0.09	0.00	0.09	0.04	0.15	0.01	0.01	0.10	0.00	0.00
142	*Pezoloma*	0.04	0.00	0.17	0.02	0.02	0.17	0.03	0.05	0.12	0.03	0.00	0.00	0.00
143	*Phaeosphaeria*	0.97	0.01	3.97	3.97	0.70	0.74	0.19	2.07	0.16	0.95	0.65	0.01	0.29
144	*Phanerochaete*	0.30	0.00	1.07	1.07	0.01	0.86	0.01	0.55	0.02	0.35	0.10	0.01	0.00
145	*Phellinus*	0.01	0.00	0.11	0.02	0.02	0.00	0.00	0.00	0.00	0.00	0.11	0.00	0.00
146	*Phialocephala*	0.05	0.00	0.19	0.00	0.19	0.07	0.09	0.01	0.07	0.03	0.05	0.00	0.01
147	*Phlebia*	0.15	0.00	0.47	0.22	0.01	0.43	0.04	0.09	0.18	0.47	0.03	0.00	0.00
148	*Phlebiopsis*	0.04	0.00	0.13	0.01	0.00	0.13	0.00	0.03	0.00	0.10	0.08	0.00	0.00
149	*Phyllosticta*	0.05	0.00	0.37	0.02	0.01	0.37	0.00	0.05	0.00	0.01	0.00	0.00	0.00
150	*Pichia*	0.09	0.00	0.84	0.01	0.00	0.00	0.05	0.00	0.84	0.00	0.00	0.00	0.02
151	*Plectosphaerella*	12.42	0.06	88.83	0.34	0.17	0.06	2.68	0.33	14.42	0.29	0.15	88.83	16.97
152	*Pleurotus*	0.05	0.00	0.26	0.00	0.05	0.02	0.03	0.00	0.26	0.02	0.01	0.02	0.09
153	*Podospora*	0.05	0.00	0.17	0.14	0.03	0.00	0.00	0.17	0.04	0.04	0.10	0.00	0.00
154	*Preussia*	0.20	0.00	0.88	0.09	0.88	0.19	0.02	0.12	0.12	0.44	0.16	0.00	0.01
155	*Psathyrella*	0.92	0.00	2.76	0.85	0.12	2.03	2.76	2.57	0.05	0.57	0.23	0.00	0.01
156	*Pseudeurotium*	0.32	0.01	1.34	0.10	1.34	0.15	0.13	0.21	0.63	0.32	0.27	0.01	0.08
157	*Pseudocenococcum*	0.02	0.00	0.11	0.00	0.11	0.00	0.00	0.00	0.05	0.00	0.00	0.00	0.00
158	*Pseudopithomyces*	0.04	0.00	0.13	0.13	0.00	0.02	0.00	0.12	0.00	0.03	0.12	0.00	0.00
159	*Purpureocillium*	0.01	0.00	0.12	0.00	0.12	0.00	0.00	0.00	0.00	0.00	0.00	0.00	0.00
160	*Pyricularia*	0.17	0.00	0.59	0.50	0.01	0.59	0.00	0.39	0.00	0.11	0.11	0.00	0.00
161	*Rachicladosporium*	0.01	0.00	0.12	0.00	0.00	0.00	0.00	0.12	0.00	0.00	0.01	0.00	0.00
162	*Resinicium*	0.05	0.00	0.34	0.01	0.01	0.34	0.00	0.00	0.00	0.13	0.04	0.00	0.00
163	*Rhexocercosporidium*	0.06	0.00	0.20	0.00	0.20	0.02	0.11	0.00	0.12	0.03	0.03	0.00	0.05
164	*Rhinocladiella*	0.15	0.00	0.68	0.12	0.68	0.00	0.01	0.09	0.28	0.18	0.15	0.00	0.02
165	*Rhizopus*	0.02	0.00	0.11	0.01	0.00	0.00	0.11	0.00	0.01	0.01	0.01	0.01	0.03

No.	Genera	Relative abundance (%)												
		Mean	Min	Max	R1_S	R1_W	R2_rural_S	R2_rural_W	R2_urban_S	R2_urban_W	R3_S	R4_S	R3_W	R4_W
166	*Rhodotorula*	0.05	0.00	0.24	0.02	0.00	0.01	0.01	0.02	0.24	0.07	0.07	0.00	0.02
167	*Rigidoporus*	0.57	0.00	4.99	0.08	0.09	0.31	0.00	0.19	0.02	4.99	0.04	0.00	0.00
168	*Roussoella*	0.04	0.00	0.12	0.09	0.05	0.01	0.00	0.06	0.01	0.12	0.03	0.00	0.01
169	*Saccharomyces*	0.03	0.00	0.14	0.00	0.14	0.00	0.02	0.01	0.03	0.00	0.00	0.01	0.13
170	*Saitozyma*	0.13	0.00	0.41	0.24	0.04	0.00	0.00	0.41	0.24	0.22	0.11	0.01	0.00
171	*Sarocladium*	0.03	0.00	0.18	0.00	0.02	0.08	0.00	0.01	0.18	0.01	0.02	0.00	0.00
172	*Schizophyllum*	0.12	0.00	0.48	0.20	0.04	0.16	0.07	0.48	0.01	0.21	0.01	0.00	0.00
173	*Schizothecium*	0.05	0.00	0.16	0.03	0.14	0.00	0.00	0.02	0.16	0.10	0.09	0.00	0.01
174	*Sclerococcum*	0.10	0.00	0.50	0.00	0.50	0.00	0.00	0.00	0.11	0.17	0.23	0.00	0.00
175	*Scopulariopsis*	0.03	0.00	0.12	0.00	0.08	0.01	0.12	0.00	0.01	0.02	0.00	0.00	0.04
176	*Scopuloides*	0.02	0.00	0.13	0.01	0.00	0.13	0.02	0.02	0.01	0.05	0.00	0.00	0.00
177	*Setomelanomma*	0.14	0.01	0.48	0.11	0.48	0.02	0.04	0.10	0.18	0.24	0.18	0.01	0.03
178	*Solicoccozyma*	0.04	0.00	0.18	0.05	0.18	0.00	0.00	0.03	0.06	0.06	0.02	0.00	0.00
179	*Sphaerulina*	0.03	0.00	0.34	0.00	0.00	0.00	0.00	0.00	0.00	0.00	0.34	0.00	0.00
180	*Spizellomyces*	0.06	0.00	0.33	0.00	0.25	0.00	0.33	0.00	0.02	0.00	0.00	0.00	0.00
181	*Stachybotrys*	0.07	0.00	0.63	0.01	0.03	0.03	0.01	0.63	0.02	0.01	0.01	0.00	0.01
182	*Stagonospora*	0.08	0.00	0.48	0.03	0.48	0.01	0.03	0.00	0.21	0.01	0.04	0.00	0.01
183	*Staphylotrichum*	0.04	0.00	0.29	0.00	0.04	0.00	0.00	0.00	0.29	0.01	0.01	0.00	0.00
184	*Subulicystidium*	0.04	0.00	0.28	0.02	0.28	0.02	0.01	0.02	0.01	0.01	0.02	0.00	0.01
185	*Symmetrospora*	0.36	0.00	3.51	0.00	3.51	0.02	0.00	0.00	0.03	0.01	0.00	0.00	0.03
186	*Talaromyces*	0.11	0.00	0.41	0.13	0.11	0.09	0.08	0.00	0.21	0.02	0.41	0.00	0.08
187	*Tetracladium*	0.38	0.00	1.76	0.08	1.76	0.08	0.06	0.18	0.87	0.57	0.17	0.00	0.08
188	*Thanatephorus*	0.03	0.00	0.17	0.02	0.03	0.03	0.00	0.17	0.00	0.01	0.00	0.00	0.00
189	*Thelebolus*	0.11	0.00	0.51	0.00	0.51	0.04	0.26	0.00	0.12	0.13	0.05	0.00	0.02
190	*Thermoascus*	0.09	0.00	0.65	0.00	0.06	0.00	0.06	0.00	0.05	0.01	0.00	0.05	0.65
191	*Thermomyces*	0.13	0.02	0.53	0.09	0.04	0.02	0.27	0.12	0.02	0.09	0.03	0.04	0.53
192	*Tomentella*	0.04	0.00	0.20	0.04	0.01	0.00	0.01	0.08	0.01	0.20	0.09	0.00	0.00
193	*Torula*	0.14	0.01	0.48	0.17	0.48	0.25	0.03	0.14	0.23	0.02	0.04	0.01	0.07
194	*Trametes*	0.48	0.01	2.20	0.18	0.14	2.20	1.01	0.24	0.32	0.15	0.26	0.01	0.26
195	*Trechispora*	0.10	0.00	0.35	0.03	0.26	0.03	0.15	0.35	0.03	0.07	0.03	0.00	0.05
196	*Trichoderma*	0.28	0.01	1.19	0.20	0.30	0.01	0.17	0.35	1.19	0.26	0.19	0.01	0.14
197	*Trichophaea*	0.04	0.00	0.20	0.03	0.00	0.00	0.00	0.03	0.00	0.20	0.17	0.00	0.00
198	*Trichosporon*	0.02	0.00	0.10	0.05	0.00	0.00	0.02	0.01	0.01	0.01	0.00	0.10	0.00
199	*Tricladium*	0.06	0.00	0.30	0.02	0.30	0.00	0.02	0.04	0.06	0.13	0.07	0.00	0.00
200	*Trimmatostroma*	0.03	0.00	0.18	0.03	0.04	0.03	0.01	0.00	0.18	0.00	0.00	0.00	0.00
201	*Typhula*	0.13	0.00	0.55	0.46	0.01	0.04	0.04	0.55	0.00	0.13	0.03	0.00	0.00
202	*Verrucaria*	0.11	0.00	0.41	0.14	0.14	0.13	0.00	0.11	0.10	0.41	0.10	0.00	0.00
203	*Volvopluteus*	0.01	0.00	0.12	0.00	0.00	0.00	0.00	0.12	0.00	0.00	0.00	0.00	0.00
204	*Wallemia*	0.07	0.02	0.21	0.02	0.07	0.03	0.11	0.02	0.21	0.05	0.03	0.13	0.03
205	*Wickerhamomyces*	0.02	0.00	0.16	0.00	0.16	0.00	0.05	0.00	0.02	0.00	0.00	0.00	0.00
206	*Xanthothecium*	0.01	0.00	0.11	0.00	0.11	0.00	0.00	0.00	0.02	0.00	0.00	0.00	0.00
207	*Xylaria*	0.09	0.00	0.32	0.32	0.01	0.01	0.01	0.31	0.00	0.15	0.08	0.00	0.00

No.	Genera	Relative abundance (%)												
		Mean	Min	Max	R1_S	R1_W	R2_rural_S	R2_rural_W	R2_urban_S	R2_urban_W	R3_S	R4_S	R3_W	R4_W
208	*Xylodon*	0.22	0.00	1.23	0.38	0.02	1.23	0.02	0.07	0.12	0.31	0.04	0.00	0.00
209	*Yamadazyma*	0.02	0.00	0.11	0.03	0.11	0.00	0.00	0.02	0.02	0.00	0.00	0.00	0.00
210	*Zasmidium*	0.12	0.01	0.52	0.04	0.15	0.52	0.18	0.02	0.09	0.07	0.04	0.01	0.07
211	*Zopfiella*	0.05	0.00	0.21	0.06	0.21	0.00	0.00	0.02	0.12	0.01	0.00	0.00	0.03
212	*Other identified*	2.50	0.19	4.01	3.39	4.01	3.46	1.28	2.53	3.19	3.79	2.18	0.19	1.00
213	*Unidentified*	15.72	0.77	22.24	21.46	22.24	15.30	15.83	20.36	12.88	21.79	15.50	0.77	11.11

Table S5. The bacterial phyla with relative abundances >0.1% in at least one group.

No.	Genera	Relative abundance (%)												
		Mean	Min	Max	R1_S	R1_W	R2_rural_S	R2_rural_W	R2_urban_S	R2_urban_W	R3_S	R3_W	R4_S	R4_W
1	Acidobacteria	1.86	0.06	5.44	0.32	0.53	0.06	3.00	1.13	3.22	0.55	5.44	0.38	4.00
2	Actinobacteria	4.02	1.04	8.52	1.07	2.54	1.04	7.57	1.42	4.37	4.17	8.08	1.45	8.52
3	Armatimonadetes	0.08	0.00	0.26	0.05	0.01	0.00	0.09	0.04	0.09	0.07	0.26	0.00	0.13
4	Bacteroidetes	7.90	1.97	18.87	1.97	10.98	3.85	6.44	3.84	18.87	5.86	12.43	2.42	12.29
5	Chloroflexi	1.01	0.09	2.68	0.21	0.33	0.09	1.44	0.61	1.64	0.40	2.52	0.19	2.68
6	Cloacimonetes	0.12	0.00	1.12	0.01	0.00	1.12	0.01	0.00	0.01	0.04	0.00	0.02	0.00
7	Crenarchaeota	0.02	0.00	0.15	0.00	0.00	0.15	0.00	0.00	0.01	0.00	0.00	0.00	0.00
8	Cyanobacteria	0.31	0.01	1.85	0.05	0.06	0.01	0.12	0.09	1.85	0.39	0.2	0.04	0.31
9	Deferribacteres	0.06	0.00	0.3.	0.00	0.09	0.00	0.04	0.00	0.30	0.00	0.09	0.00	0.03
10	Deinococcus-Thermus	0.12	0.01	0.26	0.04	0.01	0.03	0.08	0.04	0.26	0.23	0.23	0.06	0.20
11	Elusimicrobia	0.04	0.00	0.15	0.01	0.00	0.01	0.03	0.04	0.05	0.02	0.15	0.01	0.09
12	Entotheonellaeota	0.02	0.00	0.11	0.00	0.00	0.00	0.04	0.01	0.00	0.02	0.06	0.00	0.11
13	Epsilonbacteraeota	4.05	0.16	11.12	0.86	1.09	0.16	10.5	0.57	6.38	0.96	11.12	1.10	7.73
14	Euryarchaeota	0.37	0.04	1.56	0.18	0.05	1.56	0.06	0.23	0.04	1.02	0.09	0.41	0.07
15	Firmicutes	13.86	4.75	25.74	4.75	21.95	8.36	25.74	6.22	13.6	14.2	15.07	11.97	16.77
16	Fusobacteria	0.16	0.01	0.38	0.03	0.06	0.14	0.38	0.04	0.13	0.01	0.36	0.04	0.35
17	Gemmatimonadetes	0.40	0.01	1.08	0.06	0.05	0.01	0.82	0.19	0.59	0.09	1.01	0.06	1.08
18	Halanaerobiaeota	0.43	0.00	3.77	0.00	3.77	0.03	0.01	0.00	0.44	0.03	0.01	0.02	0.02
19	Latescibacteria	0.03	0.00	0.09	0.01	0.00	0.00	0.05	0.02	0.04	0.00	0.08	0.02	0.09
20	Nitrospirae	0.08	0.01	0.15	0.05	0.01	0.05	0.14	0.13	0.06	0.03	0.14	0.01	0.15
21	Patescibacteria	0.11	0.01	0.54	0.01	0.03	0.05	0.07	0.05	0.04	0.03	0.54	0.02	0.27
22	Planctomycetes	0.81	0.05	2.68	0.15	0.17	0.05	1.04	0.54	0.79	0.25	2.68	0.07	2.41
23	Proteobacteria	61.91	34.8	89.61	89.61	56.34	82.52	39.5	83.05	44.92	70.18	34.8	81.35	36.88
24	Rokubacteria	0.11	0.00	0.28	0.02	0.03	0.00	0.28	0.13	0.05	0.03	0.24	0.06	0.26
25	Spirochaetes	0.05	0.00	0.13	0.01	0.00	0.13	0.07	0.00	0.03	0.01	0.08	0.00	0.11
26	Tenericutes	0.24	0.07	0.63	0.13	0.07	0.35	0.12	0.58	0.12	0.63	0.09	0.07	0.24
27	Thaumarchaeota	0.44	0.02	1.84	0.13	0.02	0.03	0.90	0.34	0.29	0.18	0.64	0.02	1.84
28	Verrucomicrobia	1.14	0.07	3.01	0.14	1.63	0.07	1.21	0.48	1.35	0.39	3.01	0.19	2.93
29	WPS-2	0.03	0.00	0.18	0.01	0.01	0.00	0.01	0.00	0.18	0.01	0.05	0.00	0.01
30	Other identified	0.15	0.05	0.41	0.05	0.05	0.11	0.21	0.06	0.17	0.06	0.41	0.06	0.33
31	unidentified	0.09	0.00	0.16	0.06	0.09	0.03	0.04	0.16	0.11	0.13	0.14	0	0.11

Table S6. The bacterial genera with relative abundances >0.1% in at least one group.

No.	Genera	Relative abundance (%)												
		Mean	Min	Max	R1_S	R1_W	R2_rural_S	R2_rural_W	R2_urban_S	R2_urban_W	R3_S	R3_W	R4_S	R4_W
1	*[Eubacterium] coprostanoligenes group*	0.21	0.05	0.39	0.18	0.05	0.12	0.08	0.17	0.39	0.31	0.15	0.31	0.34
2	*[Eubacterium] eligens group*	0.02	0.00	0.11	0.00	0.00	0.00	0.01	0.00	0.00	0.00	0.11	0.05	0.01
3	*[Eubacterium] fissicatena group*	0.03	0.00	0.16	0.00	0.06	0.00	0.01	0.00	0.16	0.00	0.03	0.01	0.00
4	*[Ruminococcus] gnavus group*	0.03	0.00	0.12	0.01	0.04	0.00	0.01	0.01	0.01	0.01	0.07	0.12	0.06
5	*[Ruminococcus] torques group*	0.10	0.01	0.21	0.15	0.02	0.01	0.05	0.13	0.16	0.06	0.13	0.21	0.05
6	*AAP99*	0.06	0.00	0.32	0.00	0.00	0.00	0.07	0.00	0.12	0.00	0.32	0.00	0.05
7	*Acetobacter*	0.05	0.00	0.18	0.00	0.00	0.12	0.04	0.00	0.00	0.00	0.12	0.00	0.18
8	*Acholeplasma*	0.05	0.00	0.32	0.05	0.00	0.32	0.00	0.01	0.00	0.09	0.00	0.03	0.00
9	*Achromobacter*	0.07	0.01	0.46	0.02	0.01	0.46	0.03	0.01	0.06	0.03	0.02	0.01	0.01
10	*Acidibacter*	0.49	0.07	1.63	0.35	0.07	0.78	0.09	0.44	0.35	1.63	0.31	0.73	0.18
11	*Acidothermus*	0.05	0.00	0.26	0.00	0.05	0.00	0.03	0.01	0.26	0.00	0.12	0.03	0.03
12	*Acinetobacter*	0.60	0.17	1.46	0.39	0.52	0.17	0.89	0.25	0.47	0.53	0.69	0.67	1.46
13	*Actinobacillus*	0.07	0.00	0.33	0.00	0.00	0.00	0.22	0.00	0.01	0.01	0.33	0.03	0.11
14	*Actinomycetospora*	0.02	0.00	0.10	0.04	0.01	0.00	0.01	0.01	0.00	0.10	0.02	0.02	0.03
15	*Adhaeribacter*	0.02	0.00	0.11	0.01	0.00	0.00	0.03	0.01	0.03	0.01	0.02	0.00	0.11
16	*Aerococcus*	0.04	0.00	0.19	0.00	0.00	0.00	0.03	0.00	0.00	0.03	0.10	0.00	0.19
17	*Aeromonas*	0.10	0.00	0.59	0.00	0.09	0.03	0.02	0.01	0.59	0.05	0.11	0.03	0.04
18	*Afipia*	0.41	0.00	2.45	0.01	0.01	0.01	2.45	0.02	0.44	0.07	0.61	0.00	0.51
19	*Agathobacter*	0.06	0.00	0.26	0.00	0.00	0.00	0.08	0.01	0.02	0.02	0.26	0.22	0.03
20	*Aggregatibacter*	0.14	0.00	0.80	0.02	0.01	0.00	0.80	0.05	0.02	0.02	0.19	0.03	0.32
21	*Agromyces*	0.03	0.00	0.12	0.00	0.02	0.00	0.04	0.00	0.06	0.02	0.12	0.01	0.04
22	*Akkermansia*	0.34	0.00	1.44	0.00	1.44	0.00	0.26	0.01	0.56	0.11	0.47	0.00	0.50
23	*Algoriphagus*	0.01	0.00	0.10	0.00	0.00	0.00	0.00	0.01	0.02	0.10	0.00	0.01	0.00
24	*Alicycliphilus*	0.09	0.00	0.32	0.32	0.00	0.00	0.04	0.09	0.01	0.10	0.07	0.07	0.15
25	*Alistipes*	0.34	0.01	0.78	0.50	0.31	0.01	0.19	0.53	0.78	0.35	0.32	0.15	0.28
26	*Aliterella CENA595*	0.08	0.00	0.57	0.01	0.00	0.00	0.00	0.02	0.57	0.03	0.02	0.00	0.14

No.	Genera	Relative abundance (%)												
		Mean	Min	Max	R1_S	R1_W	R2_rural_S	R2_rural_W	R2_urban_S	R2_urban_W	R3_S	R4_S	R3_W	R4_W
27	*Allobaculum*	0.15	0.00	0.62	0.00	0.36	0.00	0.17	0.00	0.62	0.00	0.13	0.00	0.23
28	*Alloprevotella*	0.15	0.00	0.57	0.03	0.35	0.00	0.17	0.08	0.57	0.05	0.13	0.00	0.14
29	*Allorhizobium-Neorhizobium-Pararhizobium-Rhizobium*	0.57	0.10	2.80	0.13	0.42	2.80	0.18	0.15	0.76	0.41	0.52	0.10	0.24
30	*Altererythrobacter*	0.05	0.00	0.12	0.02	0.02	0.00	0.09	0.02	0.12	0.06	0.09	0.01	0.12
31	*Anaerophaga*	0.03	0.00	0.22	0.00	0.22	0.00	0.00	0.00	0.04	0.00	0.00	0.00	0.00
32	*Aquabacterium*	0.14	0.01	0.45	0.01	0.16	0.03	0.24	0.03	0.08	0.17	0.20	0.04	0.45
33	*Arcobacter*	0.05	0.00	0.38	0.00	0.38	0.01	0.00	0.01	0.07	0.00	0.00	0.00	0.01
34	*Arthrobacter*	0.04	0.00	0.18	0.00	0.02	0.00	0.05	0.03	0.18	0.01	0.03	0.01	0.06
35	*Asinibacterium*	0.01	0.00	0.10	0.00	0.00	0.10	0.00	0.00	0.00	0.01	0.00	0.00	0.00
36	*Aureimonas*	0.02	0.00	0.10	0.03	0.02	0.00	0.02	0.02	0.00	0.10	0.01	0.00	0.00
37	*Azoarcus*	0.01	0.00	0.10	0.00	0.00	0.00	0.00	0.00	0.00	0.00	0.10	0.00	0.00
38	*Bacillus*	1.91	0.04	13.62	0.05	0.04	0.04	13.62	0.12	0.06	0.22	2.55	0.23	2.18
39	*Bacteroides*	1.70	0.46	4.28	0.51	1.81	0.46	0.91	0.75	4.28	2.29	3.27	1.09	1.61
40	*Bdellovibrio*	0.03	0.00	0.10	0.01	0.02	0.00	0.01	0.03	0.02	0.05	0.10	0.00	0.05
41	*Bifidobacterium*	0.21	0.00	0.61	0.02	0.14	0.00	0.61	0.05	0.19	0.14	0.30	0.03	0.60
42	*Blastocatella*	0.03	0.00	0.13	0.01	0.00	0.00	0.05	0.01	0.01	0.03	0.06	0.00	0.13
43	*Blastococcus*	0.03	0.00	0.13	0.00	0.00	0.00	0.03	0.01	0.13	0.06	0.03	0.00	0.08
44	*Blautia*	0.19	0.01	0.37	0.06	0.21	0.01	0.22	0.08	0.22	0.08	0.37	0.31	0.33
45	*Bosea*	0.04	0.01	0.11	0.02	0.02	0.01	0.03	0.01	0.11	0.08	0.06	0.01	0.08
46	*Bradyrhizobium*	1.30	0.63	2.67	1.25	1.08	1.15	1.19	1.22	1.50	1.49	0.63	2.67	0.77
47	*Brevibacterium*	0.03	0.00	0.18	0.00	0.18	0.01	0.01	0.01	0.02	0.05	0.01	0.02	0.03
48	*Brevundimonas*	0.54	0.07	3.04	0.57	0.07	0.35	0.19	0.53	0.16	3.04	0.28	0.08	0.17
49	*Bryobacter*	0.08	0.00	0.20	0.02	0.04	0.00	0.14	0.05	0.18	0.05	0.20	0.03	0.15
50	*Burkholderia-Caballeronia-Paraburkholderia*	0.05	0.00	0.26	0.00	0.05	0.00	0.03	0.01	0.26	0.00	0.08	0.02	0.04
51	*Butyricimonas*	0.04	0.00	0.14	0.00	0.00	0.00	0.07	0.01	0.14	0.00	0.09	0.01	0.06
52	*Caldicoprobacter*	0.25	0.00	0.80	0.20	0.00	0.40	0.00	0.29	0.02	0.80	0.01	0.72	0.01
53	*Calothrix PCC-6303*	0.01	0.00	0.10	0.00	0.00	0.00	0.00	0.00	0.10	0.00	0.03	0.00	0.00
54	*Candidatus Methanofastidiosum*	0.02	0.00	0.17	0.00	0.00	0.17	0.00	0.00	0.00	0.00	0.00	0.00	0.00

No.	Genera	Relative abundance (%)												
		Mean	Min	Max	R1_S	R1_W	R2_rural_S	R2_rural_W	R2_urban_S	R2_urban_W	R3_S	R4_S	R3_W	R4_W
55	*Candidatus Methanoplasma*	0.06	0.00	0.35	0.03	0.03	0.35	0.00	0.03	0.03	0.10	0.00	0.05	0.00
56	*Candidatus Nitrocosmicus*	0.08	0.00	0.31	0.02	0.01	0.00	0.15	0.11	0.03	0.06	0.11	0.01	0.31
57	*Candidatus Nitrososphaera*	0.06	0.00	0.33	0.00	0.00	0.00	0.16	0.01	0.01	0.01	0.06	0.00	0.33
58	*Candidatus Solibacter*	0.07	0.00	0.23	0.03	0.02	0.00	0.08	0.03	0.16	0.03	0.23	0.03	0.05
59	*Candidatus Udaeobacter*	0.40	0.02	1.38	0.06	0.05	0.02	0.62	0.29	0.13	0.06	1.25	0.11	1.38
60	*Candidatus Xiphinematobacter*	0.04	0.00	0.13	0.01	0.01	0.00	0.04	0.04	0.13	0.01	0.10	0.01	0.11
61	*Caulobacter*	0.08	0.00	0.28	0.00	0.13	0.00	0.09	0.00	0.06	0.03	0.24	0.01	0.28
62	*Celerinatantimonas*	0.03	0.00	0.30	0.00	0.30	0.00	0.00	0.00	0.03	0.00	0.00	0.00	0.00
63	*Cetobacterium*	0.05	0.00	0.13	0.00	0.05	0.13	0.08	0.00	0.08	0.00	0.08	0.00	0.08
64	*Chitinophaga*	0.03	0.00	0.11	0.00	0.09	0.00	0.02	0.00	0.11	0.01	0.04	0.01	0.04
65	*Christensenellaceae R-7 group*	0.05	0.02	0.15	0.02	0.02	0.03	0.03	0.03	0.02	0.10	0.06	0.07	0.15
66	*Chromohalobacter*	0.40	0.00	3.67	0.00	3.67	0.00	0.02	0.00	0.30	0.00	0.01	0.00	0.02
67	*Chroococcidiopsis PCC 7203*	0.05	0.00	0.48	0.01	0.00	0.00	0.00	0.00	0.48	0.00	0.00	0.00	0.01
68	*Chryseobacterium*	0.06	0.01	0.14	0.01	0.06	0.01	0.04	0.05	0.06	0.14	0.05	0.06	0.07
69	*Chthoniobacter*	0.10	0.00	0.32	0.03	0.03	0.00	0.09	0.04	0.16	0.02	0.32	0.00	0.30
70	*Citrobacter*	0.08	0.00	0.38	0.10	0.00	0.03	0.00	0.09	0.00	0.38	0.10	0.09	0.00
71	*Clostridium sensu stricto 1*	0.21	0.00	0.46	0.12	0.05	0.00	0.46	0.14	0.23	0.35	0.25	0.05	0.40
72	*Clostridium sensu stricto 12*	0.09	0.00	0.42	0.01	0.01	0.01	0.07	0.01	0.00	0.04	0.23	0.16	0.42
73	*Cobetia*	0.04	0.00	0.39	0.00	0.39	0.00	0.00	0.00	0.02	0.00	0.00	0.00	0.00
74	*Collinsella*	0.02	0.00	0.11	0.02	0.00	0.00	0.02	0.01	0.11	0.02	0.03	0.00	0.02
75	*Comamonas*	0.06	0.00	0.32	0.05	0.03	0.32	0.02	0.01	0.00	0.13	0.04	0.04	0.02
76	*Conexibacter*	0.06	0.00	0.25	0.00	0.02	0.01	0.10	0.01	0.08	0.00	0.25	0.01	0.12
77	*Coriobacteriaceae UCG-002*	0.03	0.00	0.13	0.00	0.12	0.00	0.02	0.00	0.13	0.00	0.00	0.00	0.00
78	*Craurococcus*	0.04	0.00	0.31	0.01	0.03	0.00	0.01	0.00	0.31	0.01	0.04	0.01	0.02
79	*Deinococcus*	0.08	0.01	0.17	0.01	0.01	0.03	0.07	0.01	0.17	0.17	0.17	0.05	0.13
80	*Delftia*	0.09	0.00	0.30	0.12	0.00	0.09	0.02	0.09	0.00	0.15	0.30	0.08	0.08

No.	Genera	Relative abundance (%)												
		Mean	Min	Max	R1_S	R1_W	R2_rural_S	R2_rural_W	R2_urban_S	R2_urban_W	R3_S	R4_S	R3_W	R4_W
81	*Desulfovibrio*	0.07	0.00	0.18	0.00	0.10	0.03	0.08	0.01	0.14	0.11	0.07	0.00	0.18
82	*Devosia*	0.08	0.02	0.15	0.04	0.08	0.03	0.06	0.04	0.15	0.15	0.14	0.02	0.12
83	*Dialister*	0.04	0.00	0.14	0.01	0.00	0.00	0.06	0.01	0.00	0.01	0.14	0.02	0.09
84	*Dongia*	0.03	0.00	0.14	0.00	0.06	0.00	0.01	0.01	0.14	0.00	0.06	0.01	0.02
85	*Dubosiella*	0.12	0.00	0.59	0.00	0.10	0.00	0.17	0.00	0.59	0.19	0.07	0.02	0.08
86	*Duganella*	0.02	0.00	0.11	0.00	0.02	0.00	0.01	0.00	0.02	0.00	0.11	0.00	0.01
87	*Ellin6055*	0.05	0.00	0.16	0.01	0.00	0.00	0.16	0.03	0.09	0.06	0.09	0.00	0.07
88	*Ellin6067*	0.11	0.01	0.47	0.02	0.05	0.02	0.16	0.05	0.20	0.01	0.47	0.07	0.10
89	*Enhydrobacter*	0.05	0.01	0.11	0.03	0.02	0.01	0.07	0.01	0.02	0.11	0.11	0.05	0.10
90	*Enterobacter*	0.56	0.04	1.05	0.24	0.20	0.04	0.63	0.69	1.00	1.05	0.84	0.37	0.57
91	*Enterococcus*	0.18	0.05	0.50	0.10	0.08	0.10	0.05	0.14	0.50	0.39	0.29	0.09	0.11
92	*Enterorhabdus*	0.04	0.00	0.11	0.01	0.08	0.00	0.06	0.03	0.09	0.11	0.00	0.00	0.05
93	*Erwinia*	0.02	0.00	0.19	0.00	0.00	0.00	0.05	0.00	0.00	0.00	0.19	0.00	0.00
94	*Erysipelatoclostridium*	0.05	0.00	0.16	0.14	0.04	0.00	0.01	0.16	0.04	0.10	0.02	0.02	0.02
95	*Escherichia-Shigella*	0.61	0.07	2.22	0.24	0.32	0.49	0.34	0.27	2.22	0.07	0.63	0.67	0.86
96	*Faecalibacterium*	0.32	0.01	0.89	0.21	0.04	0.01	0.26	0.21	0.07	0.64	0.89	0.44	0.41
97	*Faecalibaculum*	0.11	0.00	0.38	0.00	0.38	0.00	0.13	0.00	0.36	0.00	0.04	0.00	0.14
98	*Fastidiosipila*	0.89	0.02	3.28	0.20	0.03	2.43	0.03	0.29	0.02	2.55	0.02	3.28	0.02
99	*Fermentimonas*	0.02	0.00	0.13	0.00	0.00	0.13	0.01	0.00	0.00	0.02	0.00	0.00	0.00
100	*Ferruginibacter*	0.12	0.00	0.41	0.00	0.02	0.01	0.24	0.07	0.18	0.03	0.41	0.00	0.23
101	*Flavisolibacter*	0.03	0.00	0.12	0.01	0.00	0.00	0.07	0.01	0.03	0.01	0.08	0.00	0.12
102	*Flavobacterium*	0.04	0.00	0.11	0.00	0.05	0.03	0.03	0.01	0.05	0.05	0.11	0.00	0.11
103	*Fusobacterium*	0.10	0.01	0.29	0.03	0.01	0.01	0.29	0.04	0.02	0.01	0.26	0.04	0.25
104	*Gaiella*	0.10	0.00	0.34	0.02	0.02	0.00	0.21	0.04	0.08	0.03	0.25	0.02	0.34
105	*Gallicola*	0.11	0.00	0.38	0.05	0.00	0.28	0.00	0.04	0.01	0.38	0.00	0.32	0.00
106	*Gemmatimonas*	0.08	0.00	0.31	0.01	0.01	0.00	0.12	0.04	0.12	0.01	0.31	0.01	0.15
107	*Gloeocapsa PCC-7428*	0.02	0.00	0.17	0.00	0.00	0.00	0.00	0.00	0.00	0.17	0.00	0.00	0.00
108	*Granulicella*	0.02	0.00	0.12	0.00	0.02	0.00	0.00	0.01	0.12	0.00	0.04	0.01	0.01
109	*Haemophilus*	0.22	0.02	0.71	0.07	0.06	0.02	0.71	0.02	0.28	0.07	0.38	0.29	0.31
110	*Halanaerobium*	0.42	0.00	3.77	0.00	3.77	0.00	0.01	0.00	0.44	0.00	0.01	0.00	0.02
111	*Haliangium*	0.09	0.01	0.41	0.03	0.04	0.01	0.08	0.06	0.10	0.02	0.41	0.02	0.10
112	*Halomonas*	0.37	0.00	3.28	0.00	3.28	0.00	0.02	0.00	0.36	0.01	0.02	0.00	0.03

No.	Genera	Relative abundance (%)												
		Mean	Min	Max	R1_S	R1_W	R2_rural_S	R2_rural_W	R2_urban_S	R2_urban_W	R3_S	R4_S	R3_W	R4_W
113	*Hathewaya*	0.02	0.00	0.12	0.00	0.00	0.00	0.12	0.00	0.00	0.00	0.04	0.00	0.06
114	*Helicobacter*	3.99	0.15	11.10	0.84	0.71	0.15	10.46	0.53	6.31	0.96	11.10	1.10	7.71
115	*Herbinix*	0.03	0.00	0.11	0.02	0.00	0.08	0.00	0.01	0.00	0.11	0.00	0.09	0.01
116	*HSB OF53-F07*	0.04	0.00	0.16	0.01	0.02	0.00	0.03	0.01	0.16	0.00	0.11	0.01	0.03
117	*Idiomarina*	0.06	0.00	0.57	0.00	0.57	0.00	0.02	0.00	0.05	0.00	0.01	0.00	0.00
118	*Ignatzschineria*	0.03	0.00	0.27	0.00	0.00	0.27	0.01	0.01	0.00	0.04	0.00	0.00	0.00
119	*Ileibacterium*	0.03	0.00	0.19	0.00	0.19	0.00	0.02	0.00	0.11	0.00	0.00	0.00	0.00
120	*IS-44*	0.03	0.00	0.15	0.00	0.00	0.00	0.02	0.01	0.07	0.00	0.15	0.00	0.04
121	*JGI 0001001-H03*	0.07	0.00	0.21	0.00	0.00	0.00	0.14	0.02	0.08	0.00	0.21	0.00	0.20
122	*Kosakonia*	0.02	0.00	0.12	0.02	0.02	0.00	0.00	0.04	0.00	0.12	0.00	0.00	0.00
123	*Kroppenstedtia*	0.02	0.00	0.14	0.00	0.00	0.00	0.00	0.00	0.00	0.00	0.07	0.00	0.14
124	*Lachnoclostridium*	0.09	0.00	0.21	0.02	0.14	0.00	0.06	0.06	0.21	0.07	0.20	0.07	0.11
125	*Lachnospira*	0.05	0.00	0.24	0.00	0.00	0.00	0.01	0.00	0.00	0.02	0.24	0.13	0.11
126	*Lachnospiraceae NK4A136 group*	0.24	0.00	0.77	0.03	0.48	0.00	0.19	0.14	0.77	0.14	0.16	0.07	0.42
127	*Lactobacillus*	3.50	0.22	15.72	0.22	15.72	0.80	5.31	0.85	4.13	0.74	2.44	0.26	4.57
128	*Lactococcus*	0.13	0.01	0.47	0.14	0.06	0.01	0.05	0.12	0.26	0.47	0.10	0.07	0.05
129	*Lawsonella*	0.07	0.00	0.48	0.00	0.01	0.01	0.11	0.01	0.00	0.02	0.48	0.08	0.01
130	*Leptolyngbya Es-Yyy1000*	0.02	0.00	0.23	0.00	0.00	0.00	0.00	0.00	0.23	0.01	0.00	0.01	0.00
131	*Leptolyngbya PCC-6306*	0.03	0.00	0.25	0.00	0.00	0.00	0.00	0.00	0.25	0.00	0.00	0.00	0.00
132	*Leucobacter*	0.03	0.00	0.17	0.00	0.00	0.02	0.01	0.01	0.00	0.01	0.07	0.01	0.17
133	*Luteimonas*	0.05	0.01	0.11	0.06	0.01	0.05	0.05	0.03	0.02	0.11	0.06	0.01	0.05
134	*Luteolibacter*	0.03	0.00	0.16	0.00	0.01	0.01	0.00	0.01	0.02	0.06	0.04	0.00	0.16
135	*Lysobacter*	0.06	0.01	0.21	0.01	0.03	0.01	0.11	0.03	0.21	0.03	0.07	0.01	0.12
136	*Marinobacter*	0.16	0.00	1.36	0.00	1.36	0.00	0.02	0.00	0.21	0.00	0.01	0.00	0.00
137	*Marinomonas*	0.03	0.00	0.19	0.00	0.06	0.00	0.01	0.00	0.19	0.00	0.01	0.00	0.00
138	*Marmoricola*	0.05	0.00	0.32	0.02	0.02	0.00	0.02	0.01	0.02	0.32	0.04	0.01	0.04
139	*Massilia*	0.31	0.00	2.07	0.00	0.34	0.01	0.13	0.03	2.07	0.03	0.24	0.04	0.17
140	*Megamonas*	0.06	0.00	0.16	0.14	0.00	0.00	0.01	0.16	0.00	0.07	0.08	0.07	0.05
141	*Megasphaera*	0.04	0.00	0.12	0.01	0.00	0.00	0.12	0.02	0.00	0.03	0.06	0.02	0.11
142	*Mesorhizobium*	0.15	0.00	0.55	0.17	0.02	0.12	0.13	0.12	0.25	0.55	0.08	0.00	0.07

No.	Genera	Relative abundance (%)												
		Mean	Min	Max	R1_S	R1_W	R2_rural_S	R2_rural_W	R2_urban_S	R2_urban_W	R3_S	R4_S	R3_W	R4_W
143	*Methanobacterium*	0.08	0.00	0.76	0.00	0.00	0.76	0.00	0.00	0.00	0.00	0.00	0.00	0.01
144	*Methanobrevibacter*	0.10	0.01	0.48	0.12	0.01	0.05	0.04	0.15	0.01	0.48	0.05	0.06	0.02
145	*Methanosarcina*	0.07	0.00	0.32	0.01	0.01	0.11	0.00	0.03	0.00	0.32	0.00	0.27	0.00
146	*Methanosphaera*	0.02	0.00	0.11	0.02	0.00	0.00	0.01	0.03	0.00	0.11	0.01	0.01	0.02
147	*Methylobacterium*	0.25	0.01	0.76	0.08	0.13	0.01	0.76	0.06	0.18	0.33	0.38	0.17	0.35
148	*Microvirga*	0.04	0.00	0.16	0.00	0.01	0.00	0.09	0.01	0.04	0.01	0.04	0.00	0.16
149	*MND1*	0.08	0.00	0.38	0.00	0.01	0.00	0.09	0.04	0.09	0.01	0.38	0.03	0.15
150	*Mucilaginibacter*	0.06	0.00	0.26	0.01	0.07	0.00	0.03	0.02	0.26	0.04	0.15	0.00	0.05
151	*Mucispirillum*	0.06	0.00	0.30	0.00	0.09	0.00	0.04	0.00	0.30	0.00	0.09	0.00	0.03
152	*Muribaculum*	0.08	0.00	0.36	0.00	0.28	0.00	0.05	0.01	0.36	0.01	0.05	0.00	0.03
153	*Mycobacterium*	0.07	0.01	0.19	0.01	0.05	0.05	0.06	0.02	0.11	0.05	0.11	0.01	0.19
154	*Mycoplasma*	0.13	0.00	0.52	0.06	0.00	0.00	0.02	0.51	0.04	0.52	0.05	0.04	0.05
155	*Nakamurella*	0.02	0.00	0.19	0.01	0.01	0.00	0.01	0.01	0.00	0.01	0.19	0.00	0.01
156	*Neisseria*	0.06	0.01	0.19	0.01	0.01	0.01	0.19	0.01	0.09	0.02	0.15	0.03	0.10
157	*Nitrospira*	0.07	0.01	0.15	0.05	0.01	0.05	0.14	0.13	0.06	0.03	0.13	0.01	0.15
158	*Nocardioides*	0.08	0.01	0.17	0.04	0.03	0.01	0.07	0.03	0.16	0.13	0.17	0.03	0.17
159	*Novosphingobium*	0.13	0.01	0.43	0.01	0.18	0.01	0.14	0.01	0.12	0.07	0.30	0.03	0.43
160	*Nubsella*	0.02	0.00	0.15	0.00	0.00	0.00	0.00	0.00	0.00	0.01	0.05	0.00	0.15
161	*Odoribacter*	0.07	0.00	0.36	0.00	0.07	0.00	0.08	0.02	0.36	0.01	0.05	0.01	0.11
162	*Oscillibacter*	0.05	0.00	0.17	0.02	0.10	0.00	0.04	0.03	0.17	0.01	0.04	0.01	0.08
163	*Paenibacillus*	0.03	0.00	0.11	0.02	0.01	0.00	0.03	0.01	0.04	0.11	0.03	0.00	0.02
164	*Pajaroellobacter*	0.14	0.01	0.82	0.02	0.01	0.82	0.09	0.03	0.10	0.12	0.12	0.04	0.04
165	*Pantoea*	0.12	0.00	0.54	0.01	0.47	0.00	0.03	0.03	0.54	0.06	0.07	0.01	0.03
166	*Parabacteroides*	0.25	0.01	0.77	0.03	0.63	0.01	0.17	0.08	0.77	0.03	0.42	0.06	0.26
167	*Paracoccus*	0.06	0.00	0.15	0.08	0.01	0.08	0.05	0.05	0.04	0.15	0.07	0.00	0.03
168	*Parasutterella*	0.27	0.00	0.80	0.20	0.43	0.00	0.11	0.25	0.80	0.07	0.31	0.02	0.47
169	*Pedobacter*	0.16	0.01	0.55	0.07	0.08	0.01	0.06	0.11	0.08	0.55	0.24	0.04	0.42
170	*Pedomicrobium*	0.04	0.00	0.13	0.02	0.02	0.00	0.08	0.05	0.04	0.03	0.13	0.03	0.06
171	*Pelomonas*	0.11	0.01	0.40	0.03	0.03	0.09	0.22	0.06	0.11	0.40	0.04	0.07	0.01
172	*Peptostreptococcus*	0.06	0.00	0.24	0.01	0.00	0.01	0.24	0.02	0.01	0.03	0.07	0.04	0.16
173	*Phascolarctobacterium*	0.07	0.01	0.22	0.02	0.01	0.02	0.04	0.03	0.13	0.03	0.22	0.14	0.11
174	*Phenylobacterium*	0.05	0.00	0.15	0.01	0.03	0.00	0.03	0.01	0.07	0.03	0.15	0.01	0.14

No.	Genera	Relative abundance (%)												
		Mean	Min	Max	R1_S	R1_W	R2_rural_S	R2_rural_W	R2_urban_S	R2_urban_W	R3_S	R4_S	R3_W	R4_W
175	*Phreatobacter*	0.28	0.00	1.96	0.00	0.00	0.00	1.96	0.01	0.55	0.06	0.08	0.00	0.12
176	*Phyllobacterium*	0.13	0.01	0.78	0.02	0.05	0.78	0.04	0.02	0.21	0.01	0.12	0.02	0.07
177	*Pir4 lineage*	0.04	0.00	0.11	0.00	0.01	0.01	0.07	0.03	0.08	0.01	0.06	0.00	0.11
178	*Pirellula*	0.04	0.00	0.13	0.00	0.01	0.00	0.06	0.03	0.05	0.02	0.10	0.00	0.13
179	*Plasticicumulans*	0.02	0.00	0.23	0.00	0.00	0.00	0.00	0.00	0.00	0.00	0.23	0.00	0.00
180	*PMMR1*	0.02	0.00	0.17	0.00	0.00	0.00	0.01	0.00	0.17	0.00	0.00	0.00	0.01
181	*Prevotella*	0.06	0.00	0.27	0.02	0.00	0.00	0.27	0.00	0.01	0.01	0.17	0.00	0.13
182	*Prevotella 7*	0.06	0.00	0.25	0.00	0.00	0.00	0.25	0.03	0.00	0.02	0.08	0.01	0.15
183	*Prevotella 9*	0.15	0.01	0.71	0.01	0.02	0.01	0.01	0.01	0.13	0.04	0.71	0.16	0.45
184	*Prevotellaceae Ga6A1 group*	0.04	0.00	0.23	0.00	0.00	0.00	0.02	0.01	0.05	0.00	0.11	0.00	0.23
185	*Prevotellaceae UCG-003*	0.07	0.00	0.31	0.00	0.01	0.00	0.06	0.00	0.15	0.01	0.14	0.00	0.31
186	*Promicromonospora*	0.03	0.00	0.16	0.00	0.03	0.06	0.02	0.00	0.16	0.01	0.00	0.00	0.02
187	*Proteiniphilum*	0.13	0.00	1.17	0.00	0.02	1.17	0.00	0.00	0.01	0.08	0.00	0.01	0.00
188	*Pseudarthrobacter*	0.83	0.14	2.64	0.37	0.47	0.14	2.64	0.39	0.30	1.33	1.15	0.56	0.99
189	*Pseudoalteromonas*	0.25	0.00	1.63	0.00	0.74	0.00	0.03	0.01	1.63	0.00	0.04	0.00	0.06
190	*Pseudomonas*	2.68	0.57	14.16	0.77	0.57	14.16	1.51	0.75	2.89	1.00	3.46	0.84	0.88
191	*Pseudonocardia*	0.07	0.00	0.21	0.03	0.02	0.01	0.06	0.05	0.06	0.12	0.10	0.00	0.21
192	*Pseudoxanthomonas*	0.05	0.00	0.12	0.01	0.07	0.00	0.06	0.02	0.12	0.03	0.06	0.02	0.11
193	*Psychroflexus*	0.02	0.00	0.18	0.00	0.18	0.00	0.00	0.00	0.01	0.00	0.01	0.00	0.00
194	*Ralstonia*	37.65	3.98	76.02	76.02	29.66	52.28	13.15	69.39	11.11	45.80	3.98	63.67	11.45
195	*Ramlibacter*	0.07	0.00	0.24	0.01	0.06	0.00	0.11	0.03	0.07	0.16	0.24	0.02	0.00
196	*RB41*	0.28	0.01	1.07	0.06	0.03	0.01	0.63	0.22	0.11	0.08	0.60	0.03	1.07
197	*Reyranella*	0.05	0.01	0.18	0.01	0.04	0.01	0.05	0.02	0.09	0.01	0.18	0.01	0.05
198	*Rhizobacter*	0.07	0.00	0.41	0.00	0.00	0.00	0.02	0.00	0.01	0.00	0.30	0.00	0.41
199	*Rhodococcus*	0.15	0.04	0.47	0.04	0.10	0.47	0.15	0.05	0.05	0.40	0.13	0.06	0.09
200	*Rhodocytophaga*	0.04	0.00	0.29	0.00	0.00	0.00	0.00	0.00	0.07	0.00	0.00	0.29	0.01
201	*Rhodomicrobium*	0.03	0.00	0.16	0.00	0.03	0.00	0.02	0.01	0.02	0.01	0.10	0.01	0.16
202	*Rhodoplanes*	0.10	0.00	0.46	0.01	0.03	0.00	0.08	0.01	0.02	0.01	0.35	0.00	0.46
203	*Rhodopseudomonas*	0.23	0.02	1.49	0.03	0.02	1.49	0.07	0.04	0.12	0.08	0.23	0.04	0.21
204	*Rikenellaceae RC9 gut group*	0.11	0.00	0.42	0.05	0.10	0.03	0.07	0.10	0.42	0.04	0.08	0.00	0.17

No.	Genera	Relative abundance (%)											
		Mean	Min	Max	R1_S	R1_W	R2_rural_S	R2_rural_W	R2_urban_S	R2_urban_W	R3_S	R4_S	R3_W	R4_W
205	*Romboutsia*	0.13	0.01	0.42	0.01	0.03	0.01	0.29	0.07	0.13	0.12	0.20	0.05	0.42
206	*Roseburia*	0.11	0.00	0.34	0.00	0.20	0.00	0.07	0.03	0.11	0.02	0.34	0.12	0.23
207	*Roseiarcus*	0.02	0.00	0.12	0.00	0.01	0.00	0.00	0.00	0.12	0.00	0.04	0.00	0.01
208	*Roseomonas*	0.06	0.00	0.39	0.02	0.00	0.00	0.01	0.01	0.39	0.09	0.02	0.00	0.05
209	*Rubellimicrobium*	0.10	0.00	0.63	0.02	0.01	0.00	0.08	0.02	0.63	0.05	0.05	0.00	0.11
210	*Rubritepida*	0.03	0.00	0.35	0.00	0.00	0.00	0.00	0.00	0.35	0.00	0.00	0.00	0.00
211	*Rubrobacter*	0.09	0.00	0.40	0.01	0.01	0.00	0.23	0.01	0.12	0.01	0.08	0.00	0.40
212	*Ruminiclostridium*	0.18	0.01	0.57	0.09	0.06	0.57	0.02	0.11	0.19	0.45	0.01	0.22	0.05
213	*Ruminiclostridium 1*	0.03	0.00	0.10	0.01	0.00	0.09	0.01	0.01	0.00	0.10	0.03	0.06	0.03
214	*Ruminiclostridium 9*	0.07	0.00	0.19	0.02	0.18	0.00	0.08	0.03	0.19	0.02	0.03	0.02	0.09
215	*Ruminococcaceae NK4A214 group*	0.05	0.01	0.18	0.01	0.01	0.02	0.06	0.01	0.01	0.07	0.11	0.06	0.18
216	*Ruminococcaceae UCG-002*	0.03	0.00	0.12	0.01	0.00	0.01	0.00	0.02	0.00	0.02	0.12	0.04	0.03
217	*Ruminococcaceae UCG-005*	0.04	0.00	0.14	0.05	0.01	0.03	0.05	0.03	0.05	0.05	0.04	0.00	0.14
218	*Ruminococcaceae UCG-010*	0.04	0.00	0.22	0.01	0.03	0.01	0.02	0.00	0.22	0.01	0.01	0.00	0.05
219	*Ruminococcaceae UCG-012*	0.04	0.00	0.18	0.02	0.00	0.12	0.00	0.02	0.00	0.18	0.00	0.09	0.00
220	*Ruminococcaceae UCG-014*	0.15	0.05	0.45	0.09	0.11	0.05	0.14	0.11	0.22	0.15	0.15	0.07	0.45
221	*Ruminococcus 1*	0.05	0.02	0.11	0.02	0.03	0.03	0.04	0.02	0.07	0.04	0.08	0.09	0.11
222	*Salinivibrio*	0.11	0.00	0.99	0.00	0.99	0.00	0.01	0.00	0.07	0.00	0.00	0.00	0.00
223	*Serratia*	0.08	0.00	0.75	0.00	0.00	0.00	0.02	0.01	0.00	0.02	0.03	0.75	0.02
224	*SM1A02*	0.03	0.00	0.10	0.00	0.01	0.00	0.05	0.01	0.04	0.01	0.10	0.00	0.05
225	*Solirubrobacter*	0.09	0.00	0.33	0.01	0.04	0.00	0.22	0.04	0.06	0.02	0.21	0.01	0.33
226	*Sphingobacterium*	0.08	0.02	0.24	0.12	0.03	0.06	0.02	0.12	0.03	0.24	0.06	0.04	0.12
227	*Sphingobium*	0.11	0.00	0.28	0.02	0.13	0.28	0.02	0.01	0.27	0.11	0.12	0.00	0.16
228	*Sphingomonas*	4.18	1.55	7.37	6.16	3.48	1.55	4.97	4.80	2.67	6.49	1.81	7.37	2.47
229	*Sphingopyxis*	0.03	0.00	0.14	0.00	0.01	0.00	0.05	0.02	0.14	0.02	0.02	0.00	0.02
230	*Sporomusa*	0.04	0.00	0.25	0.00	0.00	0.00	0.00	0.00	0.00	0.00	0.11	0.00	0.25
231	*Staphylococcus*	0.27	0.06	0.64	0.17	0.11	0.06	0.19	0.16	0.10	0.56	0.64	0.51	0.19
232	*Stenotrophomonas*	0.22	0.02	0.37	0.24	0.08	0.33	0.27	0.21	0.14	0.37	0.25	0.02	0.29

No.	Genera	Relative abundance (%)												
		Mean	Min	Max	R1_S	R1_W	R2_rural_S	R2_rural_W	R2_urban_S	R2_urban_W	R3_S	R4_S	R3_W	R4_W
233	*Steroidobacter*	0.05	0.00	0.15	0.00	0.06	0.01	0.04	0.01	0.09	0.01	0.09	0.00	0.15
234	*Streptococcus*	0.25	0.01	0.63	0.13	0.05	0.01	0.63	0.16	0.32	0.12	0.55	0.13	0.42
235	*Streptomyces*	0.05	0.00	0.11	0.01	0.11	0.01	0.06	0.01	0.11	0.01	0.07	0.00	0.08
236	*Subdoligranulum*	0.11	0.00	0.29	0.29	0.00	0.01	0.01	0.24	0.01	0.25	0.15	0.07	0.06
237	*Subgroup 10*	0.04	0.00	0.15	0.01	0.00	0.00	0.06	0.03	0.06	0.01	0.15	0.00	0.11
238	*Sutterella*	0.05	0.00	0.33	0.00	0.00	0.00	0.00	0.00	0.00	0.01	0.18	0.01	0.33
239	*Syntrophomonas*	0.04	0.00	0.11	0.04	0.00	0.08	0.00	0.02	0.00	0.11	0.01	0.10	0.01
240	*Terasakiispira*	0.17	0.00	1.47	0.00	1.47	0.00	0.01	0.00	0.23	0.00	0.01	0.00	0.00
241	*Terrimonas*	0.10	0.01	0.24	0.03	0.05	0.01	0.16	0.11	0.24	0.03	0.21	0.01	0.19
242	*Tetragenococcus*	0.05	0.00	0.45	0.00	0.45	0.00	0.00	0.00	0.04	0.00	0.01	0.00	0.01
243	*Thermomonas*	0.09	0.01	0.23	0.01	0.01	0.01	0.19	0.02	0.19	0.11	0.23	0.02	0.14
244	*Treponema 2*	0.04	0.00	0.13	0.01	0.00	0.13	0.07	0.00	0.03	0.01	0.07	0.00	0.11
245	*Truepera*	0.03	0.01	0.10	0.03	0.01	0.01	0.01	0.03	0.10	0.05	0.06	0.01	0.05
246	*Turicibacter*	0.03	0.00	0.11	0.02	0.00	0.00	0.06	0.01	0.02	0.03	0.06	0.01	0.11
247	*Variovorax*	0.24	0.02	1.43	0.09	0.05	1.43	0.07	0.04	0.19	0.11	0.29	0.02	0.08
248	*Veillonella*	0.06	0.00	0.23	0.01	0.01	0.00	0.23	0.01	0.04	0.03	0.12	0.08	0.10
249	*Vibrio*	0.05	0.00	0.36	0.00	0.36	0.00	0.00	0.00	0.11	0.00	0.00	0.00	0.00
250	*Vibrionimonas*	0.08	0.00	0.82	0.00	0.00	0.82	0.01	0.01	0.01	0.00	0.00	0.00	0.00
251	*Xanthobacter*	0.05	0.00	0.32	0.01	0.00	0.00	0.02	0.00	0.00	0.01	0.11	0.00	0.32
252	*Zoogloea*	0.14	0.00	0.47	0.00	0.06	0.02	0.28	0.05	0.25	0.02	0.47	0.00	0.21
253	*Other identified*	4.39	1.80	9.05	2.21	2.89	1.80	4.62	2.70	5.54	4.90	9.05	2.75	7.42
254	*Unidentified*	12.68	2.78	26.13	2.78	11.76	5.92	15.12	6.83	21.75	6.94	25.97	3.58	26.13

Table S7. The relative abundances of trophic modes of fungi in each region in summer and winter.

Trophic mode	Relative abundance (%)										
	R1_S	R2_rural_S	R2_urban_S	R3_S	R4_S	R1_W	R2_rural_W	R2_urban_W	R3_W	R4_W	Mean
Pathotroph-Saprotroph	24.3	22.4	20.5	19.6	18.6	23.9	22.4	12.3	0.6	13.1	17.77
Pathotroph-Saprotroph-Symbiotroph	15	16.1	18.8	10.3	19.1	16.1	10.3	17.3	0.4	15.7	13.91
Pathotroph-Symbiotroph	1.9	2.6	2.9	2.1	2.3	3	12	38.4	98.2	53.5	21.69
Saprotroph	55.2	56.4	53	61.1	52.8	49.8	52.9	29.7	0.8	16.9	42.86
Saprotroph-Symbiotroph	1.1	1	1.2	2.5	2.4	4.4	1.8	1	0	0.4	1.58
Symbiotroph	2.6	1.5	3.6	4.4	4.9	2.8	0.7	1.3	0	0.4	2.22

Table S8. The relative abundances of growth forms of fungi in each region in summer and winter. The numbers are relative abundances (%).

Growth form	R1_S	R2_rural_S	R2_urban_S	R3_S	R4_S	R1_W	R2_rural_W	R2_urban_W	R3_W	R4_W	Average
Agaricoid	12.56	13.00	23.96	13.95	18.46	12.48	14.79	1.88	0.12	2.11	11.33
Agaricoid-Gasteroid-	1.35	1.19	0.81	1.69	0.63	0.20	2.36	0.05	0.00	0.15	0.84
Agaricoid-Gasteroid-Secotioid	0.15	0.40	0.10	0.14	0.07	2.26	0.00	0.00	0.00	0.00	0.31
Boletoid	0.00	0.02	0.05	0.15	0.02	0.00	0.05	0.00	0.00	0.00	0.03
Clavarioid	0.06	0.13	0.22	0.66	0.52	1.21	0.06	0.06	0.00	0.01	0.29
Coralloid	0.00	0.03	0.07	0.05	0.00	0.00	0.02	0.03	0.01	0.00	0.02
Corticioid	8.29	15.03	5.81	8.83	3.56	3.73	1.33	1.13	0.02	0.37	4.81
Corticioid-Gasteroid	0.00	0.00	0.00	0.00	0.00	0.00	0.00	0.00	0.00	0.00	0.00
Corticioid-Microfungus	0.02	0.03	0.21	0.05	0.10	0.16	0.15	0.01	0.00	0.00	0.07
Corticioid-Polyporoid	0.09	1.05	0.10	0.18	0.00	0.04	0.00	0.02	0.00	0.00	0.15
Corticioid-Thallus	0.02	0.00	0.00	0.00	0.00	0.01	0.00	0.02	0.00	0.00	0.01
Facultative Yeast	0.21	0.04	0.32	0.63	0.22	0.20	0.47	0.06	0.00	0.21	0.24
Facultative Yeast-Microfungus	2.42	0.32	1.81	3.71	2.70	6.82	1.07	2.26	0.16	1.18	2.25
Facultative Yeast-Smut	0.00	0.00	0.01	0.01	0.00	0.04	0.00	0.00	0.00	0.00	0.01
Gasteroid	0.17	0.00	0.46	0.51	0.48	0.66	0.40	0.08	0.00	0.00	0.28
Gasteroid-Pezizoid	0.00	0.00	0.00	0.08	0.01	0.01	0.00	0.00	0.00	0.00	0.01
Leaf Gall-Microfungus	0.00	0.00	0.00	0.00	0.00	0.00	0.00	0.00	0.00	0.00	0.00
Microfungus	64.09	53.63	58.46	43.91	60.33	47.48	56.67	77.64	99.59	91.22	65.30
Microfungus-Thallus	0.01	0.03	0.00	0.60	1.24	1.31	0.00	0.27	0.00	0.00	0.35
Pezizoid	0.11	0.03	0.06	0.25	0.12	0.80	2.68	1.68	0.00	0.09	0.58
Polyporoid	3.88	13.15	2.38	19.86	4.51	1.95	15.39	7.05	0.06	3.27	7.15
Rust	0.00	0.00	0.00	0.00	0.31	0.00	0.00	0.00	0.00	0.00	0.03
Smut	0.09	0.04	0.04	0.00	0.00	0.00	0.00	0.00	0.00	0.00	0.02
Thallus	1.88	0.56	2.78	2.44	3.86	0.62	0.01	0.36	0.01	0.06	1.26
Tremelloid	0.07	0.60	0.16	0.61	0.17	0.02	0.00	0.03	0.00	0.01	0.17
Xylarioid	1.46	0.40	1.22	1.16	1.97	0.92	0.03	0.20	0.00	0.01	0.74
Yeast	3.08	0.32	0.97	0.53	0.71	19.09	4.51	7.19	0.03	1.32	3.78

Table S9. The 30 strongest functions predicted by FAPROTAX in the regions in summer and winter. The numbers are relative abundances (%).

	R1_S	R2_rural_S	R2_urban_S	R3_S	R4_S	R1_W	R2_rural_W	R2_urban_W	R3_W	R4_W	Average
Aerobic Ammonia Oxidation	0.04	0.01	0.12	0.06	0.04	0.04	0.50	0.22	0.87	1.49	0.34
Aerobic Chemoheterotrophy	2.43	4.80	2.36	5.13	3.43	8.58	6.11	8.68	8.18	10.63	6.03
Animal Parasites or Symbionts	0.62	0.28	0.81	1.34	1.12	1.04	8.48	6.08	9.91	9.35	3.90
Aromatic Compound Degradation	17.89	12.30	17.52	14.94	16.71	10.83	6.39	5.38	2.52	1.17	10.57
Aromatic Hydrocarbon Degradation	17.78	12.25	17.44	14.68	16.50	10.63	5.52	4.99	1.92	0.05	10.18
Chemoheterotrophy	21.07	17.76	21.15	22.72	21.79	29.10	17.64	22.59	17.90	20.77	21.25
Chloroplasts	0.32	18.88	0.29	0.92	0.23	0.64	1.34	0.67	0.37	0.51	2.42
Dark Hydrogen Oxidation	0.05	0.24	0.06	0.38	0.09	0.03	0.06	0.04	0.20	0.49	0.16
Fermentation	0.87	0.66	1.36	2.89	1.78	10.78	6.09	9.23	8.11	10.48	5.23
Fumarate Respiration	0.18	0.04	0.13	0.30	0.28	0.11	4.29	2.59	5.49	4.86	1.83
Human Associated	0.59	0.28	0.76	1.27	0.94	0.93	7.45	5.71	8.69	8.10	3.47
Human Gut	0.48	0.18	0.65	1.00	0.90	0.79	5.97	4.93	8.12	7.35	3.04
Human Pathogens All	0.30	0.13	0.25	0.56	0.34	0.23	5.82	3.38	6.13	5.67	2.28
Hydrocarbon Degradation	17.81	12.26	17.45	14.69	16.51	12.47	5.54	5.26	1.96	0.09	10.40
Intracellular Parasites	0.17	5.27	0.08	0.24	0.06	0.08	0.30	0.19	0.23	0.38	0.70
Mammal Gut	0.48	0.18	0.65	1.01	0.91	0.79	5.97	4.93	8.12	7.36	3.04
Methanol Oxidation	0.04	0.02	0.03	0.17	0.05	0.09	0.34	0.11	0.29	0.32	0.15
Methylotrophy	0.04	0.13	0.03	0.20	0.06	0.10	0.34	0.13	0.32	0.33	0.17
Nitrate Reduction	0.25	0.34	0.41	0.68	0.62	0.68	1.59	2.59	2.40	2.80	1.24
Nitrate Respiration	0.09	0.19	0.11	0.23	0.01	0.10	0.38	0.29	0.71	0.70	0.28
Nitrification	0.05	0.02	0.15	0.07	0.04	0.04	0.54	0.24	0.94	1.58	0.37
Nitrite Respiration	0.02	0.03	0.03	0.08	0.01	0.13	0.22	0.52	0.45	0.45	0.19
Nitrogen Fixation	0.31	0.26	0.31	0.52	0.69	0.38	0.51	0.76	0.41	0.78	0.49
Nitrogen Respiration	0.09	0.20	0.11	0.23	0.01	0.18	0.39	0.65	0.72	0.70	0.33
Photoautotrophy	0.01	0.00	0.02	0.13	0.01	0.02	0.07	1.03	0.28	0.54	0.21
Photoheterotrophy	0.01	0.32	0.01	0.06	0.03	0.04	0.11	0.27	0.38	0.60	0.18
Phototrophy	0.02	0.33	0.03	0.19	0.03	0.05	0.14	1.28	0.45	0.73	0.33
Plastic Degradation	0.03	0.37	0.02	0.11	0.01	0.02	0.54	0.19	0.28	0.02	0.16
Ureolysis	17.88	12.24	17.54	15.11	16.63	10.99	7.25	6.77	3.05	1.37	10.88
Xylanolysis	0.07	0.01	0.11	0.10	0.15	0.12	0.10	0.29	0.61	0.32	0.19
Others	0.51	0.86	2.02	2.92	0.64	4.31	2.45	4.41	0.66	4.63	2.34
